# Atrial ERK1/2 activation in the embryo leads to incomplete Septal closure: a novel mouse model of atrial Septal defect

**DOI:** 10.1186/s12929-017-0392-2

**Published:** 2017-11-24

**Authors:** Che-Chung Yeh, Yanying Fan, Yi-Lin Yang, Michael J. Mann

**Affiliations:** 1 Cardiothoracic Translational Research Laboratory, University of California, San Francisco, San Francisco, CA USA; 2 Division of Cardiothoracic Surgery, 500 Parnassus Avenue, Suite W420, San Francisco, CA 94143 USA

**Keywords:** Atrial septal defect, RASopathies, Mitogen-activated protein kinase, MAP kinase phosphatase, Mitogen-activated protein kinase pathway

## Abstract

**Background:**

MEK1 mutation and activated MAPK signaling has been found in patients with RASopathies and abnormal cardiac development. Previous studies have suggested that regulation of fetal MAPK signaling is essential for normal cardiac development. We investigated the effect of active MEK1 overexpression on fetal atrial septal development.

**Methods and results:**

An inducible double transgenic (DTg) mouse model was developed in which cardiac-specific fetal expression of a constitutively active form of human MEK1 (aMEK1) was induced primarily in the atrium via the withdrawal of doxycycline from the drinking water of pregnant mice. Atrial septal defect (ASD) was found in 51% (23/45) of DTg mice. Fifty-two percent (12/23) of ASD mice died before weaning, and surviving ASD mice exhibited hypertrophic hearts with enlarged right atria and decreased fractional shorting (40 ± 2% vs. 48 ± 0%, *p* < 0.05). The model mimicked human ASD in several key clinical features: severe ASD was associated with growth impairment; ASD-specific mortality was highest within the early postnatal period; despite an even distribution of ASD among the sexes, early mortality was significantly higher in males. The expression of aMEK1 and increased phosphorylation of ERK1/2 was documented via Western blot in DTg fetal hearts, with the largest increases seen in atrial tissue. In an alternative transgenic aMEK1 model with elevated atrial MKP3 expression and corresponding suppression of increases in ERK1/2 phosphorylation, animals did not develop ASD.

**Conclusion:**

This new model of ASD suggests that enhanced atrial MEK1-ERK1/2 signaling during fetal development disrupts normal atrial septation, possibly regulated by the balance of ERK1/2 phosphorylation.

**Electronic supplementary material:**

The online version of this article (10.1186/s12929-017-0392-2) contains supplementary material, which is available to authorized users.

## Background

Atrial septal defects (ASD) are among the most common types of congenital heart disease (CHD), with an estimated incidence of 56–100 per 100,000 live births [1]. While mutations in GATA4, MYH6, NKX2–5 and TBX5 genes have been linked to the abnormal septation of atrial chambers, most patients with ASD are diagnosed without known etiologic causes [2–5]. However, the risk of a secundum defect is increased in siblings of ASD patients, suggesting that an inherited molecular mechanism may play a role in abnormal atrial septation.

The function of MEK1 signaling in the adult heart has been associated with “physiologic” hypertrophy [6]. Overexpression of active MEK1 induces hypertrophic changes in adult left ventricular (LV) structure that increase cardiac function and that do not degenerate into cardiomyopathy [6]. Increased MEK1 activity also protects hearts under stress conditions such as ischemia-reperfusion and myocardial infarction [7, 8]. In contrast to this positive influence of MEK1 activity in adult hearts, recent advances in the study of RASopathies have demonstrated a potentially detrimental role for Ras-Raf-MEK-ERK signaling during embryonic/fetal heart development [9]. RASopathies occur as a cluster of syndromes with germline mutations in genes participating in the Ras-Raf-MEK-ERK kinase signaling pathway. Independent of mutations and other syndromes, CHD - including ASD - are common in RASopathies patients and are a major source of morbidity and even mortality [9]. Although mutations in human MEK1 genes have been identified in RASopathies, and the early application of a MEK1 inhibitor in animal models has been able to ameliorate associated defects [10–13], there is no direct evidence indicating how MEK1 may contribute to CHD.

We observed a very high incidence of ASD in double transgenic (DTg) mice in which expression of a constitutively active isoform of human MEK1 (aMEK1) regulated by an α-myosin heavy chain (αMHC) promoter was induced during fetal development. Although the αMHC promoter is generally used as a means to induce myocardial-specific gene expression in adult hearts, this promoter is also active in the embryonic/fetal heart with higher activity in the atrial region [14]. No previous model of relatively atrial-specific overexpression of aMEK1 in the developing fetus has been reported, nor are there reproducible, specific model.s of congenital ASD that closely mimic the human clinical scenario.

## Methods

### Animals

Hemagglutinin (HA)-tagged, constitutively active human MEK1 (aMEK1) cDNA, kindly provided by Dr. Natalie Ahn, was subcloned into the pTet-Splice vector (Invitrogen Corp.) for most of the experimentation reported, and also into the pTREtight vector (Clontech, Mountain View, CA) for validation of the transgenic model. The aMEK1 expression DNA cassettes were excised from both the pTet-Splice vector and the pTREtight vector, and each was used for pronuclear injection to generate founders carrying tetracycline responsive element regulated expression of aMEK1 gene (TAMEK). Genotyping was done by PCR using Primers 5′-GAGCTGGGGGCTGGCAATGG-3′ and 5′-CCTTGGCCTGGGTGGGGTCT-3′. Positive founders derived from each of the vectors were bred with C57BL/6 mice to obtained stable TAMEK transgenic (Tg) lines. The cardiac specific expression of tetracycline-responsive transcriptional activator (tTA) αMHC-tTA Tg mice (MH) were obtained from the Jackson Laboratory and were crossed with TAMEK mice to generate TAMEK/MH DTg mice. The tTA transgene was detected by PCR using Primers 5′-CGCTGTGGGGCATTTTACTTTAG-3′ and 5′-CATGTCCAGATCGAAATCGTC-3′. DTg mice and breeding pairs were treated with Doxycycline (DOX, 0.5 mg/ml) in drinking water for suppression of transgene expression. DOX was withdrawn from the drinking water to induce fetal aMEK1 transgene expression. Wild type and /or single Tg littermates were used as controls for DTg mice. The MEK1 transgenic mouse, in which aMEK1 is driven by the αMHC promoter without DOX regulation, was kindly provided by Dr. Jeffrey Molkentin [6]. Time mating was validated by checking the vaginal plugs in the morning and marking as 0.5 day post copulation (dpc). All animals were maintained at a 12 h light–dark cycle with ad libitum access to food and water. All procedures conformed to the Guide for the Care and Use of Laboratory Animals published by the US National Institutes of Health (NIH Publication No. 85–23, revised 1996), and were approved by the Institutional Animal Care and Use Committee of the University of California, San Francisco. Please see Additional file 1 S1 for additional details on Methods.

### Anatomy and histology analysis

Mouse hearts were arrested in diastole with i.p. injection of 1 ml of 1 M KCl in saline, formalin-fixed and embedded in paraffin. To assess the morphology of the atrial septum, the right atrial appendage was removed, and the anatomical structure of septal wall was assessed under dissection microscopy. To document ASD and possible ventricular septal defects, serial continuous sections of paraffin embedded hearts were stained with Gomori’s trichrome reagent [8]. Stained sections were reviewed for occurrence of septal defect and photographic images were acquired.

### Western blotting assay

Fetal hearts were collected from fetuses at 10.5 to 14.5 dpc; some fetal hearts were then divided into atrial and ventricular portions. Each tissue sample was homogenized in lysis buffer containing 150 mM NaCl, 50 mM Tris-HCl, 1 mM Na3VO4, 5 mM NaF, 1% NP40, and protease inhibitor cocktail tablet (Roche Diagnostics, Indianapolis, IN). Samples containing equal amounts of protein were separated by NuPAGE Novex Bis-Tris Gels (Invitrogen, Carlsbad, CA) and transferred to PVDF membranes (Invitrogen). Blots were first probed with antibodies against phospho-ERK1/2 (P-ERK1/2, rabbit monoclonal, 1:2000, #4376, Cell Signaling, Danvers, MA,) or total ERK1/2(rabbit polyclonal, 1:4000, #9102, Cell Signaling), total MEK1/2 (rabbit monoclonal, 1:4000, #9126, Cell Signaling), MKP1(rabbit polyclonal, 1:1000, SC-1102, Santa Cruz Biotech, Santa Cruz, CA), MKP3(mouse monoclonal, 1:1000, SC-1000374, Santa Cruz Biotech), tTA (mouse monoclonal anti-TetR, 1:2000, #631131, Clontech, Mountain View, CA), GAPDH (rabbit polyclonal, sc-25,778, Santa Crus Biotech) or HA (mouse monoclonal, 1:2000, #2367, Cell Signaling), and then with appropriate horseradish peroxidase-conjugated secondary antibodies (goat anti-rabbit IgG-HRP, 074–1056 and goat anti-mouse IgG-HRP, 074–1806, KPL, Gaithersburg, MD). SuperSignal West Femto Maximum Sensitivity substrate (Thermo Fisher Scientific Inc., Waltham, MA) and Immobilon Western HRP Substrate (EMD Millipore, Billerica, MA) were used for the chemiluminescent visualization of proteins. Exposed films were then subjected to density analysis using Image J software (NIH).

### Echocardiography

Transthoracic echocardiography was performed in conscious 8-week old female mice using an Acuson Sequoia 512 machine and a 13-MHz probe [8]. A two dimensional short-axis view of the left ventricle was obtained at the level of the papillary muscles. Two-dimensional M-mode tracings were also recorded. LV fractional shortening (FS) and ejection fraction (EF) were calculated using the following equation: FS (%) = 100 × (LV end of diastolic dimension - LV end of systolic dimension) /LV end of diastolic dimension; EF (%) = 100 × (end of diastolic volume - end of systolic volume)/end of diastolic volume.

### Statistics

Results are expressed as mean ± SEM. Mean values were compared by the unpaired 2-tailed Student’s t test or ANOVA. Mortality rates were compared by Fisher’s exact test. *P*-values less than 0.05 were considered statistically significant. Although we examine many potential effects, we report nominal *p*-values, without adjustment for multiple testing. Such adjustment would be focused on avoidance of one or more results with *p* < 0.05 in the case where all differences are truly zero [15–17], which is an extremely unrealistic hypothesis about the state of nature in our situation. We therefore rely on scientific judgment rather than formal adjustment methods to indicate where caution is warranted despite findings with *p* < 0.05. In addition, adjustment would require that each result detract from the others, but there are clear biological relationships among the issues that we examine, and these permit coherent sets of findings to reinforce each other rather than detract from one another [18].

## Results

### Doxycyline induced aMEK1 expression and activity in TAMEK/MH double transgenic mice (DTg)

**Fig. 1:**
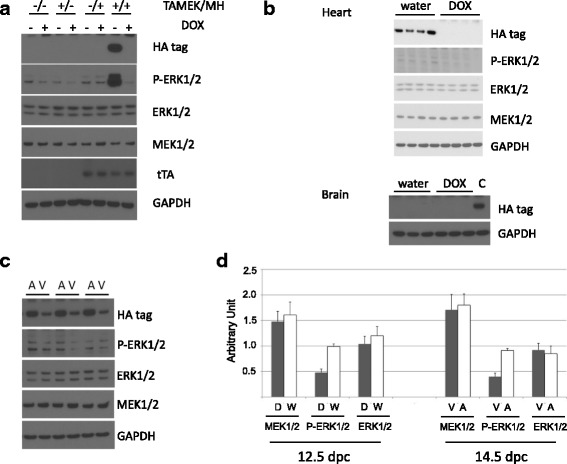
Regulation of MEK1 transgene and ERK1/2 expression. **a** Activation of the HA-tagged human aMEK1 is regulated by the expression of tTA and doxycycline (DOX) in the TAMEK/MH DTg mouse heart. **b** At 12.5 dpc, fetal hearts expressed the aMEK1 transgene when the pregnant females did not receive DOX in their drinking water. Expression of aMEK1 transgene was absent in the fetal brain. **c** At 14.5 dpc, the expression of aMEK1 transgene. And corresponding ERK1/2 phosphorylation, is higher in the atria than in the ventricles as demonstrated in paired tissues (*n* = 5). **d** Quantitation of MEK1/2, ERK1/2 and P-ERK1/2 protein from water- (W) or DOX- (D) treated mice at 12.5 dpc, and from ventricular (V) and atrial (A) tissue at 14.5 dpc. Original blots available in Additional file 5 S5

Even though αMHC expression is traditionally associated with the adult heart, previous studies have demonstrated the expression of this MHC isoform and the activity of its promoter in fetal hearts [14]. In the absence of DOX in the drinking water of pregnant mice, expression of the HA-tagged human aMEK1 transgene, dependent on tTA expression driven by the αMHC promoter, was observed in DTg fetuses. Its expression was subsequently suppressed in DTg fetuses via introduction of DOX into the drinking water of pregnant mice (Fig. 1A). Although expression of the aMEK1 transgene was detectable via identification of an otherwise absent HA tag, the level of expression achievable in the fetal heart as driven by a combination of the relatively low activities of the αMHC promoter and the tTA promoter in the fetal heart was not strong enough to yield a measurable increase in total MEK1 protein. As has been observed previously, however [19], the constitutive activity even of this relatively smaller amount of aMEK1 protein compared to the total pool of non-phosphorylated and non-activated endogenous MEK1 resulted in a measurable increase in downstream ERK1/2 activation (i.e., phosphorylation, Fig. 1A-C).

During fetal development, aMEK1 transgene expression was identified in 14.5 dpc hearts, but aMEK1 expression was absent in the fetus’ brain tissue (Fig. 1B). Furthermore, there was a differential expression pattern of aMEK1 transgene even within the developing fetal hearts. Expression of the HA-tagged aMEK1 gene, as well as increased phosphorylation of ERK1/2, was significantly higher in atrial tissue than in ventricular tissue from 14.5 dpc hearts of DTg fetuses (Fig. 1C).

### Atrial septal defect in DTg in the absence of DOX (and in the presence of upregulated ERK1/2 phosphorylation)

**Fig. 2:**
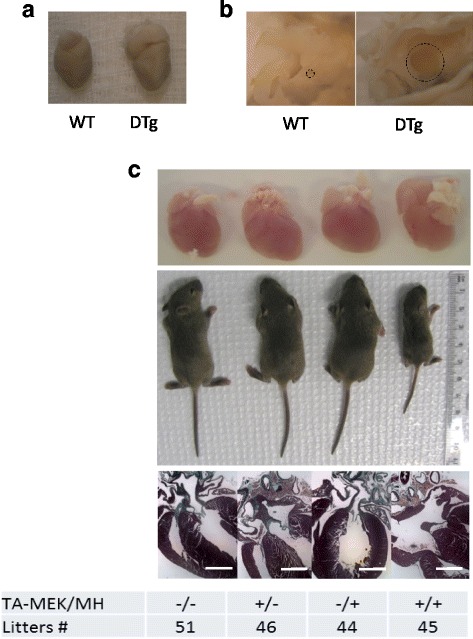
TAMEK/MH DTg mice developed ASD. **a** Heart from DOX-free DTg with enlarged right atrium compared to WT. **b** Anatomical dissection showed an unfused open foramen ovale (dashed circle) in the atrial septal wall of DTg hearts. **c** Comparison of progeny of 4 genotypes at 14-days of age reveals enlarged hearts (and in particular, enlarged right atria), growth retardation, and atrial septal defects in DTg’s. The distribution of progeny followed Mendel’s Law of segregation at birth

Hearts from a large number of DTg mice (23 out of 45) that had not been exposed to DOX in utero exhibited enlarged right atria compared to the right atria from their non-DTg littermates (Fig. 2A). Further anatomical analyses of 8 week-old hearts with hypertrophied right atria revealed the large, unfused foramen ovale connecting both atrial chambers, resembling secundum atrial septal defects (Fig. 2B, Additional file 2 S2). In contrast, only small vestigial foramena ovale were observed in WT hearts (Fig. 2B). The ASDs in DTg hearts were confirmed on whole heart histologic sections taken at 14 days of age, although no other ventricular or atrioventricular abnormalities were observed in DTg hearts, with or without in utero exposure to DOX (Fig. 2C).

### Premature mortality and compromised cardiac function in DTg

**Fig. 3:**
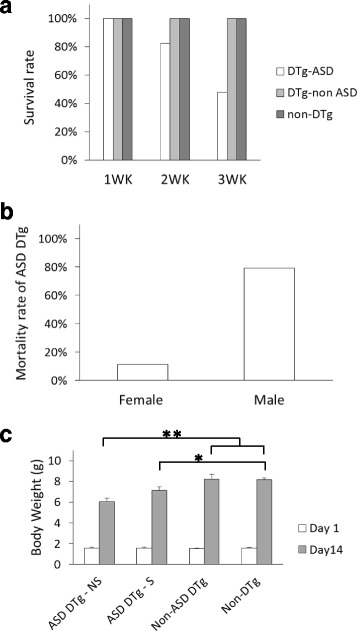
**a** Mortality in ASD mice. Fifty-one percent early postnatal mortality in ASD-affected DTg (12 out of 23 by 3 weeks of age), compared to 100% 3-week survival in the non-DTg and non-ASD DTg groups. **b** Gender difference in mortality. Male ASD mice (*n* = 14) had higher mortality than female ASD mice (*n* = 9, 79% vs 11%; *P* = 0.003). **c** Early mortality is associated with poor weight gain. There is no difference in average bodyweights among ASD DTg mice and other groups at birth. However, the average body weight at 14 days after birth is significantly lower in the ASD DTg mice. ASD DTg non-survivor (NS) group *n* = 4; ASD DTg - survivor (S) group, *n* = 6; non-ASD DTg, n = 9; non-DTg group, *n* = 40. **P < 0.01, *P < 0.05

In the absence of DOX exposure, crossbreeding of heterozygous TAMEK1 and MH mice yielded pups with four different transgenic genotypes in a classic Mendelian distribution (Fig. 2C). By postnatal day 21, however, only 73% (33/45) of the DTg pups had survived, while there was no mortality among the other three genotypes (Fig. 3A). Although the incidence of ASD was similar in male and female DTg in the early postnatal period, mortality was observed in 11/14 (79%) of male ASD-DTg’s at 21 days, compared to only 1/9 (11%) of female ASD-DTg’s at that time point (*P* = 0.003, Fig. 3B).

Even though DTg pups as a whole had similar initial birth bodyweights compared to their non-DTg littermates, postnatal growth of DTg pups subsequently diagnosed with ASD, but not DTg pups without ASD, was delayed at day 14 (6.1 ± 0.4 g for ASD-DTg vs. either 8.2 ± 0.2 g for non-DTg or 8.2 ± 0.5 g non-ASD DTg, *p* < 0.01, Fig. 2C & 3C). Among the 14-day old DTg pups with ASD, the average bodyweight of mice that survived at wean (21 days) was marginally higher than those mice that did not subsequently survive (7.1 ± 0.4 g vs 6.1 ± 0.4 g, *P* = 0.07), suggesting a link between severity of the ASD and early postnatal growth. This early growth impairment may therefore serve as a diagnostic marker for severe ASD in this mouse model.

At 8 weeks of age, the body weight difference between surviving female ASD mice and other genotype/phenotype female mice had disappeared (we included only female groups in our statistical analysis due to the insufficient number of surviving male ASD-DTg mice, Table 1), reflecting better overall compensation for ASD in surviving ASD mice. All single transgenic and WT mice survived past the time of wean. Echocardiography was performed on 8-week old female mice from the four different genotypes. Compared to the non-ASD groups, the ASD-affected female DTg mice, despite equivalent body weights, had significantly decreased LV function as reflected both in lower fractional shortening (40 ± 2% vs 48 ± 0%, *p* < 0.05) and reduced ejection fraction (67 ± 2% vs. 75 ± 0%, p < 0.05; Table 1, Additional file 3 S3).

**Table 1:** Echocardiographic measurements of LV function in 8-week old mice bearing different transgenic genotypes

Genotype (TAMEK/MH)	−/−	+/−	−/+	+/+
BW(g)	22 ± 1	21 ± 0	21 ± 0	22 ± 0
EF(%)	75 ± 0	75 ± 0	76 ± 0	64 ± 2*
FS(%)	48 ± 0	48 ± 0	48 ± 0	37 ± 2*
IVSd (mm)	0.51 ± 0.05	0.53 ± 0.06	0.51 ± 0.05	0.48 ± 0.05
IVSs (mm)	0.65 ± 0.11	0.66 ± 0.11	0.65 ± 0.10	0.60 ± 0.09
LVAWd (mm)	0.80 ± 0.06	0.83 ± 0.06	0.79 ± 0.05	0.78 ± 0.06
LVAWs (mm)	1.15 ± 0.09	1.15 ± 0.08	1.11 ± 0.08	1.10 ± 0.08
LVIDd (mm)	3.70 ± 0.08	3.74 ± 0.09	3.67 ± 0.07	4.11 ± 0.09*
LVIDs (mm)	2.51 ± 0.13	2.55 ± 0.10	2.48 ± 0.09	2.51 ± 0.10*
n	5	5	5	5

*BW* body weight, *EF* ejection fraction, *FS* fractional shortening, *IVS* interventricular septum, *LVAW* left ventricular anterior wall, *LVID* left ventricular internal dimension; +/+ group includes ASD mice only; please see Additional file 6 S6 for data from +/+ non-ASD mice. *P < 0.05

### Differential phosphorylation of ERK1/2 in the developing atria of two different aMEK1 transgenic models correlates to differential development of ASD

**Fig. 4:**
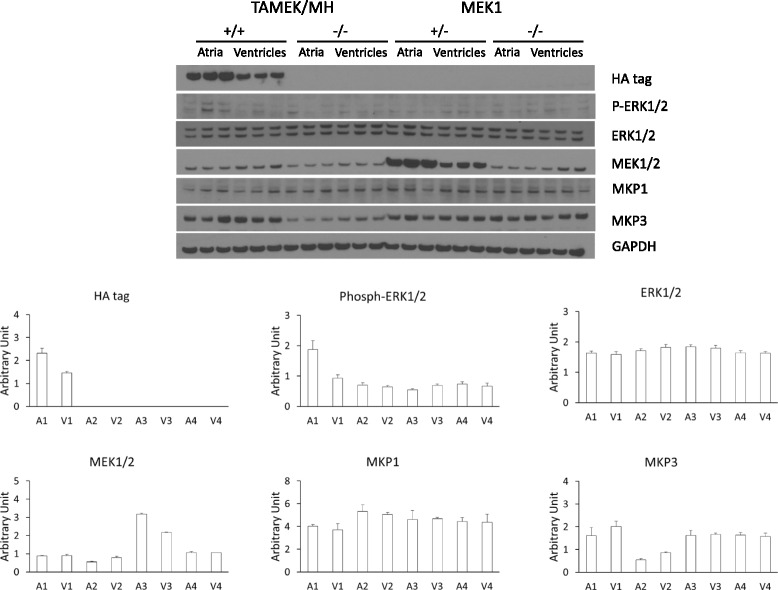
Regulation of the MEK-ERK signaling pathway in 14.5 dpc hearts from two different aMEK1 transgenic models. Despite evidence of aMEK1 expression in the atria of both TAMEK/MH DTg and MEK1 single Tg mice, increased ERK1/2 phosphorylation was documented only in the DTg. *P < 0.05. P-ERK1/2 = Phospho-ERK1/2; A1/V1 = pooled atrial and ventricular tissues, respectively, from TAMEK/MH DTg; A2/V2 = pooled atrial and ventricular tissues, respectively, from TAMEK/MH WT sibling controls; A3/V3 = pooled atrial and ventricular tissues, respectively, from MEK1 single Tg; A4/V4 = pooled atrial and ventricular tissues, respectively, from MEK1 WT sibling controls. Original blots available in Additional file 7 S7

In addition to the DTg model of inducible, cardiac-specific aMEK1 expression described here, our laboratory has also studied an alternative transgenic model, the MEK1 Tg mouse, in which cardiac-specific expression of aMEK1 is constitutive under the αMHC promoter and is not affected by DOX. In contrast to the inducible TAMEK/MH DTg model, the constitutively expressing MEK1 Tg mouse (a generous gift from the Jeffrey Molkentin laboratory) did not develop ASD or other congenital cardiac defects. We evaluated fetal transgene expression in the MEK1 Tg model, and did observe aMEK1 expression in 14.5 dpc fetal hearts as reflected by an increase in total MEK1 protein (Fig. 4). As in the TAMEK/MH DTg model, the 14.5 dpc expression of aMEK1 in the constitutively expressing transgenic was also higher in the atria than in the ventricles. However, the pattern of atrial phosphorylation of ERK1/2 was markedly different in the two transgenic models. Although aMEK1 expression in adult MEK1 Tg hearts is associated with increased ERK1/2 expression and phosphorylation [6], neither atrial nor ventricular phosphorylation of ERK1/2 was significantly increased in heart tissues from constitutively expressing fetal 14.5 dpc MEK1 Tg mice compared to their 14.5 dpc WT siblings (Fig. 4). This difference in downstream ERK1/2 activation despite similar fetal atrial aMEK1 expression in the MEK1 Tg and our novel TAMEK/MH DTg mice correlated with the different phenotypes observed with regard to congenital ASD development, as ASD was only observed in DTg mice in which atrial ERK1/2 phosphorylation was greater than that of both ventricular tissue from the same hearts and heart tissues from non-DTg littermates,.

In order to evaluate the possible roles of integration site and the use of different transgene expression vectors in the different molecular and clinical phenotypes observed in TAMEK/MH DTg and MEK1 Tg mice, we developed a second inducible model using the pTREtight vector. We observed a similar incidence and clinical impact of ASD in this second DTg line (Additional file 4, S4).

We also observed another interesting difference between the inducible TAMEK/MH DTg and the constitutively expressing MEK1 mouse with regard to MEK/ERK regulation. Expression of the negative regulator of ERK phosphorylation/activation, MAP kinase phosphotase 3 (MKP3) [19] was significantly higher in the atria and ventricles of 14.5 dpc TAMEK/MH DTg mice than in cardiac tissues from their WT siblings (Fig. 4, Additional file 4 S4, *P* < 0.05). However, a similarly high level of MKP3 expression was observed in both (constitutively expressing) MEK1 Tg mice and in their respective WT siblings (Fig. 4). In contrast, the expression of MKP1, which also regulates p38 and JNK phosphorylation [20], was similar among atrial and ventricular tissues from all four groups of mice (Fig. 4).

## Discussion

The severity of human ASD differs drastically among affected individuals, ranging from life-threatening to symptomless. Although ASD is one of the most common congenital heart defects, this clinical variability in phenotype makes identification of factors that interrupt normal atrial septal development even more difficult. There are several genetic mouse models that exhibit defects in atrial septation. Among the most often reported are models of functionally impaired or heterozygous null NKX2–5 and GATA4, based largely on early associations of mutations in these genes for these transcription factors with human ASD and other congenital defects [2, 3, 21–23]. These models of transcription factor disruption, however, have proven to be extremely complex, as their mechanisms involve disruption not only of the target transcription factor but also impaired expression of other related transcription factors such as TBX5 and MEF2c [21]. As a result, changes in numerous downstream effectors regulated by these multiple transcription regulators are at play. Confounding the mechanistic analyses of these models is their frequent association with extracardiac abnormalities, and the fact that they are often embryonic lethal and demonstrate compound septal defects involving both atria and ventricles.

In contrast, the model described here of isolated congenital ASD offers a relatively focused mechanistic pathway for future study; it is directed to a well-characterized signaling pathway rather than a complex of pleiotropic upstream transcription factors. In addition to the mechanistic complexity of previously reported genetic models of ASD, the perinatal characteristics of those models do not closely mimic the clinical findings in human ASD patients. Our novel model of isolated ASD, however, resembles human ASD in the following important ways: severe ASD is associated with growth impairment; ASD-specific mortality is highest within the early postnatal period; although the incidence of ASD is evenly distributed among the sexes, this early mortality is significantly higher in males ([24, 25]; Table 2).

**Table 2:** Similarities between human ASD and the MEK1-mediated mouse ASD model

	Human ASD	MEK1 ASD mouse model
Gender distribution at birth	51% male	56% male
Gender dependent long term survival ate	65% survivors were female (>5 years)	89% survivors were female (21 days)
Major mortality stage	Early, first year after birth (75% of total death)	Early, before weaning (100% of death till 8wk old)
Growth impairment	Weight and length	Weight and length

Our current mouse model likely reflects an important role of Ras-MEK-MAPK signaling in the development of RASopathy syndromes, including atrial septal defects. In fact, we have observed for the first time that upregulation of MEK-ERK signaling in an atrial-specific manner, resulting in higher atrial ERK1/2 phosphorylation, is associated with abnormal atrial septation. Furthermore, by comparing two aMEK1 transgenic mouse models, we substantiated the potential role for site-specific ERK1/2 activation in the development of ASD.

Since MAPK signaling pathways can be regulated by various stimuli, such as growth factors, hormones, exogenous chemicals and even mechanical stresses [26–30], our findings also suggest that genetic-independent, aberrant activation of ERK1/2 during pregnancy may lead to the sporadic occurrence of ASD.

The mechanism by which upregulated ERK1/2 activation is absent in the fetal atria of the constitutively expressing MEK1 mouse, despite fetal cardiac aMEK1 expression driven by the αMHC promoter, remains unclear. One explanation for differences in phenotype between the two transgenic models studied may be related to differences in integration site and the use of different transgene expression vectors in their generation. However, we observed a similar incidence and clinical impact of ASD in a second DTg line derived via a distinct integration using an alternative vector.

**Fig. 5:**
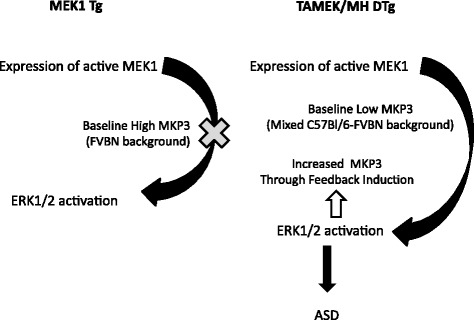
Proposed role of baseline MKP3 expression in determining ERK1/2 activation and, in turn, putative induction of ASD phenotype in transgenic mice with expression of constitutively active MEK1 (see Discussion)

It is also possible that the difference in genetic backgrounds between the double transgenic (C57Bl/6-FVBN mix in both lines studied) and single transgenic (pure FVBN) mouse models may modulate the downstream effects of aMEK1 transgene expression. The importance of genetic background as a possible regulator of congenital defects has been demonstrated previously in the *Nkx2–5* transgenic mouse [31]. Heterozygous *Nkx2–5* knockout mice in the inbred strain background C57Bl/6 frequently developed atrial and ventricular septal defects, whereas the incidences of these defects were substantially reduced in the *Nkx2–5*(+/−) progeny outcrossed to the strains FVB/N or A/J [31]. Since the pure FVBN-derived single Tg MEK1 and its WT siblings both had higher fetal cardiac MKP3 expression than the WT siblings of the C57Bl/6-FVBN-derived DTg or control C57Bl/6 mice, it is possible that the expression level of MKP3 is related to this difference in genetic backgrounds. Higher pre-existing MKP3 levels in the fetal atria of MEK1 single Tg hearts may have suppressed activation of ERK1/2, thereby resulting in resistance to ASD despite aMEK1 transgene expression (Fig. 5). The higher levels of MKP3 observed in the DOX-withdrawn DTg may, in turn, reflect a feedback loop that is not as effective as high constitutive MKP3 expression in controlling ERK1/2 activation. In fact, previous studies have indicated that MKP3-mediated suppression of ERK activation is required for proper development of the kidneys, neural plate and limb bud mesenchyme [32–34]. A similar mechanism may be at play to explain why lower levels of αMHC-driven aMEK1 expression in fetal MEK1 Tg hearts did not exhibit the increased ERK1/2 phosphorylation seen in adult MEK1 Tg hearts with significantly higher αMHC-driven aMEK1 expression. Studies in MKP3-null mouse lines derived from mice with different genetic backgrounds have also demonstrated variable influences on development even with similar increases in ERK phosphorylation [35, 36]. The results reported here do suggest that the fine tuning of ERK1/2 signaling via MKP3 expression levels at specific locations within the developing heart may be essential for the proper organ and tissue development [37].

Developmental defects in the brain have also been observed in models of upstream ERK activation via Braf Q241R or Neurofibromatosis type 1 gene overexpression. Treatment with MEK inhibitors was found to correct developmental defects in these experimental animals [11–13, 38]. While ERKs are known to be preferentially activated both in the regions of the primitive brain and in the heart during early development, our new evidence underscores the importance of carefully regulated MAPK signaling during fetal development. Any dysregulation or over-activation of this pathway that leads to an increase in ERK1/2 phosphorylation may impact the normal development of specific organ structure or function.

## Conclusions

Our data indicate that the regulation of MAPK signaling is essential for normal development of the atrial septum. Specifically, atrial upregulation of activated ERK1/2 was associated with failure of normal septal closure. The TAMEK1/MH DTg mouse is the first ASD-specific model that closely mimics the clinical manifestations of human congenital ASD, and therefore may serve as a novel tool to elucidate both the development of human fetal septal defects as well as possible therapeutic interventions. Furthermore, our data also begin to suggest that modifiers of MAPK signaling in fetal hearts, such as MKP3 and other internal/environmental factors, should be further evaluated to better understand the complex, multifactorial development of ASD and other congenital defects.

## Additional files

Additional file 1: S1 Methods. (DOCX 34 kb)

Additional file 2: S2 Figure. Comparison of progeny of 4 genotypes at 8 weeks of age showed atrial septal defect in DTg (+/+). (PPTX 1404 kb)

Additional file 3: S3 Figure. Survived ASD affected DTg (+/+, from line #25) and its non- DTg siblings at 8 weeks old. (PPTX 206 kb)

Additional file 4: S4 Figure Anatomical dissection revealed ASD in two independent DTg lines at 8 weeks old. A. line #25, derived from expression sequence using pTet-Splice vector; B. line #8, derived from expression sequence using pTREtight vector. C. MEK1/2, P-ERK1/2, ERK1/2, MKP1, and MKP3 protein levels as detected by Western blot in atrial (A) and ventricular (V) tissue from 14.5 dpc DTg (+/+) and WT (−/−) littermates from line #8 (C57BI/6-FVBN mixed background) and MKP1, and MKP3 from control pure C57BI/6 mice. D. Survival among DTg mice from line #8 with (*n* = 11) and without (*n* = 12) ASD, and among non-DTg littermates (*n* = 66). (PDF 1225 kb)

Additional file 5: S5. Original blots in fig. 1. (PPTX 1641 kb)

Additional file 6: S6 Table Echocardiographic measurements of LV function in the 8 weeks old DTg mice (ASD and non-ASD). (DOCX 14 kb)

Additional file 7: S7. Original blots in fig. 4. (PPTX 1028 kb)

## Abbreviations

A: Atria
aMEK1: Active form of human MEK1
ASD: Atrial septal defects
CHD: Congenital heart disease
DOX: Doxycycline
Dpc: Day post copulation
DTg: Double transgenic (DTg)
EF: Ejection fraction
ERK1/2: Mitogen-activated protein kinase ½
FS: Fractional shortening
HA: Hemagglutinin
i.p.: Intraperitoneal
LV: Left ventricular
MEK1: Mitogen-activated protein kinase kinase 1
MH: αMHC-tTA transgenic mouse
MKP: MAP kinase phosphatase
P-ERK1/2: Phospho-ERK1/2
TAMEK: Transgenic mouse with tetracycline transactivator regulated aMEK1 gene
TetR: Tetracycline repressor protein
Tg: Transgenic mouse
tTA: Tracycline-responsive transcriptional activator
V: Ventricle
α-MHC: α-myosin heavy chain
