# Supplementary material for: Atrial ERK1/2 activation in the embryo leads to incomplete Septal closure: a novel mouse model of atrial Septal defect
Source: J Biomed Sci. 2017 Nov 24;24:89. doi: 10.1186/s12929-017-0392-2 (PMC5702213; doi:10.1186/s12929-017-0392-2)
Supplement: Supplementary file 1 — S1 Methods. (DOCX 34 kb) [file 12929_2017_392_MOESM1_ESM.docx]

**S1 Methods**

**Plasmid construction**

The pMCL-HA-MAPKK1-R4F plasmid carrying human active MEK1 mutant (Δ31-51, S218E, S222D) was provided by Dr. N.A. Ahn, this constitutively active MEK1 had basal activities up to 400 times greater than that of the unphosphorylated wild-type kinase (1). The HA-tagged human active MEK1 cDNA sequence was released by XbaI-HindIII restriction digestion and subcloned into HindIII site of pTet-Splice vector (Invitrogen Corp.) and pTREtight vector (Clontech, Mountain View, CA). Oritation and sequence of inseted MEK1 cDNA in the vectors validated by XbaI-BamHI or NheI-HindIII restriction maps and sequenceing.

**DNA Preparation for Microinjection**

The pTet-MEK1 and pTREtight-MEK1 plasmid DNAs were preparedred using EndoFree Plasmid kit (Qiagen, Valencia, CA). Digest 100 ug of each vector with restriction digestion enzymes XhoI and BglI that cutted off MEK1 expression cassette out of cloning vectors overnight. The digested vectors were separated on a 0.8% agarose gel, the corrrseponding bands were excised and extracted by Qiagen QIAEX II Gel Extraction Kit (Qiagen, Valencia, CA). Extracted DNA was ethanol precipitated for a couple of hours with 2.5 volume of 100%ethanol on ice. Centrifuged at maximum speed (13,500rpm) for 10 minutes to pellet DNA. DNA was washed with 70% ethanol and centrifuged at maximum speed for 5 minutes. Repeated wash step and allowed pellets to airdry. DNA pellets were resuspended in sterile PBS to a concentration of 1ug/ul. Run a 0.8% gel with 500 ng samples to check for linearization and verify DNA concentration. DNA was diluted to 50ng per μl of linearized DNA for producing transgenic mice. Pronuclear microinjection of DNA for producing transgenic mice was provided by the Gladstone Institutes at UCSF.

**Mice**

Hemagglutinin (HA)-tagged, constitutively active human MEK1 (aMEK1) cDNAwas subcloned into the pTet-Splice vector for most of the experimentation reported, and also into the pTREtight vector for validation. The excised aMEK1 expression cassettes were used for pronuclear injection to generate founders, which was performed the Gladstone Institutes. Postive founders were bred with C57BL/6 mice to obtained stable TA-MEK Tg lines. The αMHC-tTA Tg (MH) mice were obtained from the Jackson Laboratory (2) and were crossed with TA-MEK mice to generate DTg mice. DTg mice and breeding pairs were treated with Doxycycline (Dox, 0.5 mg/ml) in drinking water for suppression of transgene expression. Wild type and /or single Tg littermates were used as controls to DTg mice. The MEK1 transgenic mouse (Tg) was kindly provide by Dr. Jeffrey Molkentin (3). Time mating was validated by checking the vaginal plugs in the morning and marking as 0.5 day post copulation (dpc). All animals were maintained at a 12 h light–dark cycle with ad libitum access to food and water. All procedures conformed to the Guide for the Care and Use of Laboratory Animals published by the US National Institutes of Health (NIH Publication No. 85-23, revised 1996), and were approved by the Institutional Animal Care and Use Committee of the University of California, San Francisco.

**Genotyping**

Toe tips from 5-10 days old pups or tails from fetus are placed in 1.5 ml tube with 0.2 ml tissue lysis buffer (10U Proteinase K; 50 mM KCl; 10 mM Tris-HCl, pH8.3; 2.5 mM MgCl_2_; 0.1 mg/ml gelatin; 0.45% Nonidey P40; 0.45% Tween20). Incubate overnight at 55 °C to completely digest sample tissue and release genomic DNA. After completion of digestion, Proteinase K was inactivated at 100 °C for 10 minutes; the solution was centrifuged at 12,000 rpm for 3 min to remove precipitate. The cleared supernatant containing genomic DNA was used for genotyping. Genotyping was done by polymerase chain reaction (PCR) using GoTaq Green Master Mix (Promega, Madison, WI) and primers 5′-GAGCTGGGGGCTGGCAATGG-3′ and 5′-CCTTGGCCTGGGTGGGGTCT-3′ for TA-MEK Tg, with expected PCR product size of 650bp. The PCR products were differentiated in 1.2% agarose gels to identify specific transgene fragments. Postive TA-MEK Tg founders were bred with C57BL/6 mice to obtained stable TA-MEK Tg lines. αMHC-tTA Tg (MH) mice were obtained from the Jackson Laboratory and were crossed with TA-MEK mice to generate DTg mice. The tTA transgene was detected by PCR using Primers 5′-CGCTGTGGGGCATTTTACTTTAG-3′ and 5′-CATGTCCAGATCGAAATCGTC-3′ with expected PCR product size of 500bp. The MEK1 transgenic mouse (Tg) was genotyped using Primers 5′-CACTTTCTGCTTCTGGGTGAG-3′ and 5′-CAGGGAAGTGGTGGTGTAG-3′, with expected PCR product size of 300bp. The PCR was performed in GeneAmp PCR System 9700 (Applied Biosystems, Foster City, CA).

**Mointoring phenotypes in DTg mice**

The parameters monitored included morbidity, mortality, development, anatomic/histologic abnormalities, teratogenicity, and lifespan in addition to those listed in the chart below. Pups were assessed for their growth rate and in the presence of underweight or underdeveloped, pups were weighed daily and the weight recorded. Pups were assessed for their growth rate and those that were underweight or underdeveloped were weighed daily and the weight recorded.

**Anatomy and histology analysis**

Hearts from 2-8 weeks old mice were arrested in diastole with i.p. injection 0.04 ml/g body weight of 1M KCl in saline. The arrested hearts were perfused with iced cold PBS fro 5 min to remove blood in the system, and dissected, weighted and fixed in 10% zinc-formalin (Thermo Fisher Scientific Inc., Waltham, MA) . To assess the morphology of the atrial septum, the right atrial appendage was removed from fixed hearts, and the anatomical structure of septal wall was assessed and recorded under SZ61 stereo microscope and Camdia C-5060 camera (Olympus America Inc., Center Valley, PA). Some formalin-fixed hearts were dehydrated in serial 70-100% ethanol and xylenes, and embedded in paraffin. To document ASD and possible ventricular septal defects, serial of continuous 7μm sections of paraffin embedded hearts were stained with Gomori’s trichrome reagent (4,5). Tissue sections were first deparaffinized in xylenes for 3 times and rehydrated in serial 100-70% ethanol and water. Rehydrated sections were fix in Bouin’s solution (Ricca Chemical, Arlington, TX) for 1 hour at 56°C. Sections were washed under running water for 5 min to remove excess Bouin’s stain. Immersed sections in Weigert’s hematoxylin (Thermo Fisher Scientific Inc.) for 10 minutes, and washed with tap water until the water is clear. Immersed sections in Gomori trichrome stain (Thermo Fisher Scientific Inc.) for 10 minutes and differentiated the staining with a few dips in the 0.2% acetic acid. Rinsed sections in deionized water for 30 seconds and dehydrated section in serial 70-100% ethanol and xylenes. Mounted coverslip onto each glass slide with Shandon-Mount (Thermo Fisher Scientific Inc.).Stained sections were reviewed under Imager.Z2 microscope (Carl Zeiss Microscopy, Jena, German) for incidence of septal defect and respective images were acquired by attached AxioCam HRm (Carl Zeiss Microscopy).

**Western blotting assay**

The timed pregnant female MH mice were euthanized by 1M KCl and isoflurane overdose at 14.5 dpc. Uterus with futuses were immediately dissected and place in ice-cold PBS. All tissue harvest procedures were perform in a PBS covered petri dish over ice. Each fetus was removed from uteru and washed 3 times with ice-cold PBS to remove blood. To dissect the fetal hearts and brains, individual fetus was placed under SZ61 stereo microscope to operate the microdissection, and its corresponding tail tissue was collected for genotyping. Fetal hearts were collected from fetuses at 14.5 dpc; some fetal hearts were then divided into atrial and ventricular portions. Each tissue sample was homogenized in lysis buffer containing 150mM NaCl, 50mM Tris-HCl, 1mM Na3VO4, 5mM NaF, 1% NP40, and protease inhibitor cocktail tablet (Roche Diagnostics, Indianapolis, IN) and then centrifuged at 16,000 g for 20 min at 4ºC to remove tissue debris. The supernatant was collected and total protein was quantified using a bicinchoninic acid (BCA) assay (Thermo Fisher Scientific Inc.).

To quantitate the protein concentrations, , pipette 20μL of each standard or unknown sample replicate into a well of a 96-well microplate, add 180μL of the working reagent to each well and mix plate thoroughly on a plate shaker for 30 seconds to 1 minute, cover plate and incubate at 37°C for 30 minutes. Plates were read at 562 nM in the BioTek EL800 Plate Reader (BioTek Instruments, Winooski, VT). For Western analyses, 5 μg of each tissue sample was prepared by adding 1/4 final volume 4XNuPAGE sample buffer and 1/10 volume reducing agent (stabilized DTT from Invitrogen), heating at 10min at 70 ºC, then separated in NuPAGE 4-12% Bis-Tris Midi gels (Invitrogen, Carlsbad, CA; 5 μg of protein loaded per lane). After electrphorsis, separated samples were transferred from gel onto a PVDF membranes (Invitrogen, [Carlsbad, CA](https://www.google.com/search?biw=1920&bih=986&q=Carlsbad+California&stick=H4sIAAAAAAAAAOPgE-LUz9U3MDNLKUxS4gAxi0zK87S0spOt9POL0hPzMqsSSzLz81A4VhmpiSmFpYlFJalFxQDermitQwAAAA&sa=X&sqi=2&ved=0ahUKEwiBmcSMttPNAhVbGGMKHe63BnIQmxMIkgEoATAO)) by electroblotting in a Bio-Rad Criterion^TM^ Blotter (Hercules, CA) at 75V constant voltage for 45min. Membranes were blocked in 5% milk in TBS with 0.1% Tween-20 (TBST) for 1h and then incubated overnight at 4ºC with the following primary antibodies in 5% milk TBST: phospho- ERK1/2 (rabbit monoclonal, 1:2000, #4376, Cell Signaling, Danvers, MA,) or total ERK1/2(rabbit polyclonal, 1:4000, #9102, Cell Signaling), total MEK1/2 (rabbit monoclonal, 1:4000, #9126, Cell Signaling), MKP1(rabbit polyclonal, 1:1000, SC-1102, Santa Cruz Biotech, Santa Cruz, CA), MKP3(mouse monoclonal, 1:1000, SC-1000374, Santa Cruz Biotech), tTA (mouse monoclonal anti-TetR, 1:2000, #631131, Clontech, Mountain View, CA), GAPDH (rabbit polyclonal, sc-25778, Santa Crus Biotech) or HA (mouse monoclonal, 1:2000, #2367, Cell Signaling). Membranes were subsequently incubated with their respective HRP-conjugated secondary antibodies in 5% milk TBST (1:50,000; goat polyclonal anti-rabbit IgG-HRP, 074-1056 and goat polyclonal anti-mouse IgG-HRP, 074-1806, KPL, Gaithersburg, MD). SuperSignal West Femto Maximum Sensitivity substrate (Thermo Fisher Scientific Inc.) and Immobilon Western HRP Substrate (EMD Millipore, Billerica, MA) was used for the chemiluminescent visualization of proteins. Exposed films were then subjected to density analysis using Image J software (NIH)..

Echocardiography

The day before the procedure, chest hairs were removed by using Nair hair remover (Church & Dwight Co.,Inc. Princeton, NJ). 2-D and M-mode images are obtained using Accuson echocardiography equipment (Sequoia 512, Acuson, Mountain View, CA) with a 13 MHz transducer in conscious mice restrained in DECAPICONE (Braintree Scientific, MA) mouse restrainer. Ultrasound transmission gel (Parker Laboratories, Inc, Fairfield, NJ) was applied in the space between DECAPCONE and chest skin and the restrained mouse was placed in a left lateral decubitus position on a flat plateform. The transducer was layered with ultrasound transmission gel and placed gently at chest wall. With fine adjustments, a two-dimensional short-axis view of the left ventricle was obtained at the level of the papillary muscles. M-mode tracings were recorded through the anterior and posterior LV walls at a paper speed of 100 mm/s. During echocardiographic analysis, digital images were retrieved and analyzed on the same echocardiography system. Anterior and posterior wall thicknesses (end-diastolic and end-systolic) and left ventricle (LV) internal dimensions were measured using a modification of the American Society for Echocardiography leading edge method from at least three consecutive cardiac cycles on the M-mode tracings. LV fractional shortening (FS) and ejection fraction (EF) were calculated using the following equation: FS (%) = 100 × (LV end of diastolic dimension - LV end of systolic dimension) / LV end of diastolic dimension; EF (%) = 100 × (end of diastolic volume - end of systolic volume)/ end of diastolic volume.

Statistics

Results are expressed as mean ± SEM. Mean values were compared by the unpaired 2-tailed Student’s t test or ANOVA. Mortality rates were compared by Fisher’s exact test. P-values less than 0.05 were considered statistically significant.

**References**

1. Mansour SJ, Matten WT, Hermann AS, Candia JM, Rong S, Fukasawa K, Vande Woude GF, Ahn NG. Transformation of mammalian cells by constitutively active MAP kinase kinase. *Science.* 1994;265:966-70.
2. Yu Z, Redfern CS, Fishman GI. Conditional transgene expression in the heart. *Circ Res*. 1996; 79: 691–697.
3. Bueno OF, De Windt LJ, Tymitz KM, Witt SA, Kimball TR, Klevitsky R, Hewett TE, Jones SP, Lefer DJ, Peng CF, Kitsis RN, Molkentin JD. The MEK1-ERK1/2 signaling pathway promotes compensated cardiac ypertrophy in transgenic mice. *EMBO J.* 2000;19:6341-6350.
4. GOMORI G. A rapid one-step trichrome stain. Am J Clin Pathol. 1950Jul;20(7):661-4.
5. Yeh CC, Malhotra D, Yang YL, Xu Y, Fan Y, Li H, Mann MJ. MEK1-induced physiological hypertrophy inhibits chronic post-myocardial infarction remodeling in mice. J Cell Biochem. 2013;114:47-55.
6. Towbin H, Staehelin T, Gordon J. [Electrophoretic transfer of proteins from polyacrylamide gels to nitrocellulose sheets: procedure and some applications](https://www.ncbi.nlm.nih.gov/pmc/articles/PMC411572). Proceedings of the National Academy of Sciences USA. 1979;76 : 4350–54.
7. Mahmood T, Yang PC. Western blot: technique, theory, and trouble shooting. N Am J Med Sci. 2012;4:429-34.
8. Sahn DJ, DeMaria A, Kisslo J, Weyman A. Recommendations regarding quantitation in M-mode echocardiography: results of a survey of echocardiographic measurements. Circulation. 1978;58:1072–83.
9. Kanno S, Lerner DL, Schuessler RB, Betsuyaku T, Yamada KA, Saffitz JE, Kovacs A. Echocardiographic evaluation of ventricular remodeling in a mouse model of myocardial infarction. J Am Soc Echocardiogr. 2002;15:601-9.
