# Supplementary material for: Atrial ERK1/2 activation in the embryo leads to incomplete Septal closure: a novel mouse model of atrial Septal defect
Source: J Biomed Sci. 2017 Nov 24;24:89. doi: 10.1186/s12929-017-0392-2 (PMC5702213; doi:10.1186/s12929-017-0392-2)
Supplement: Supplementary file 2 — S2 Figure. Comparison of progeny of 4 genotypes at 8 weeks of age showed atrial septal defect in DTg (+/+). (PPTX 1404 kb) [file 12929_2017_392_MOESM2_ESM.pptx]

## Slide 1
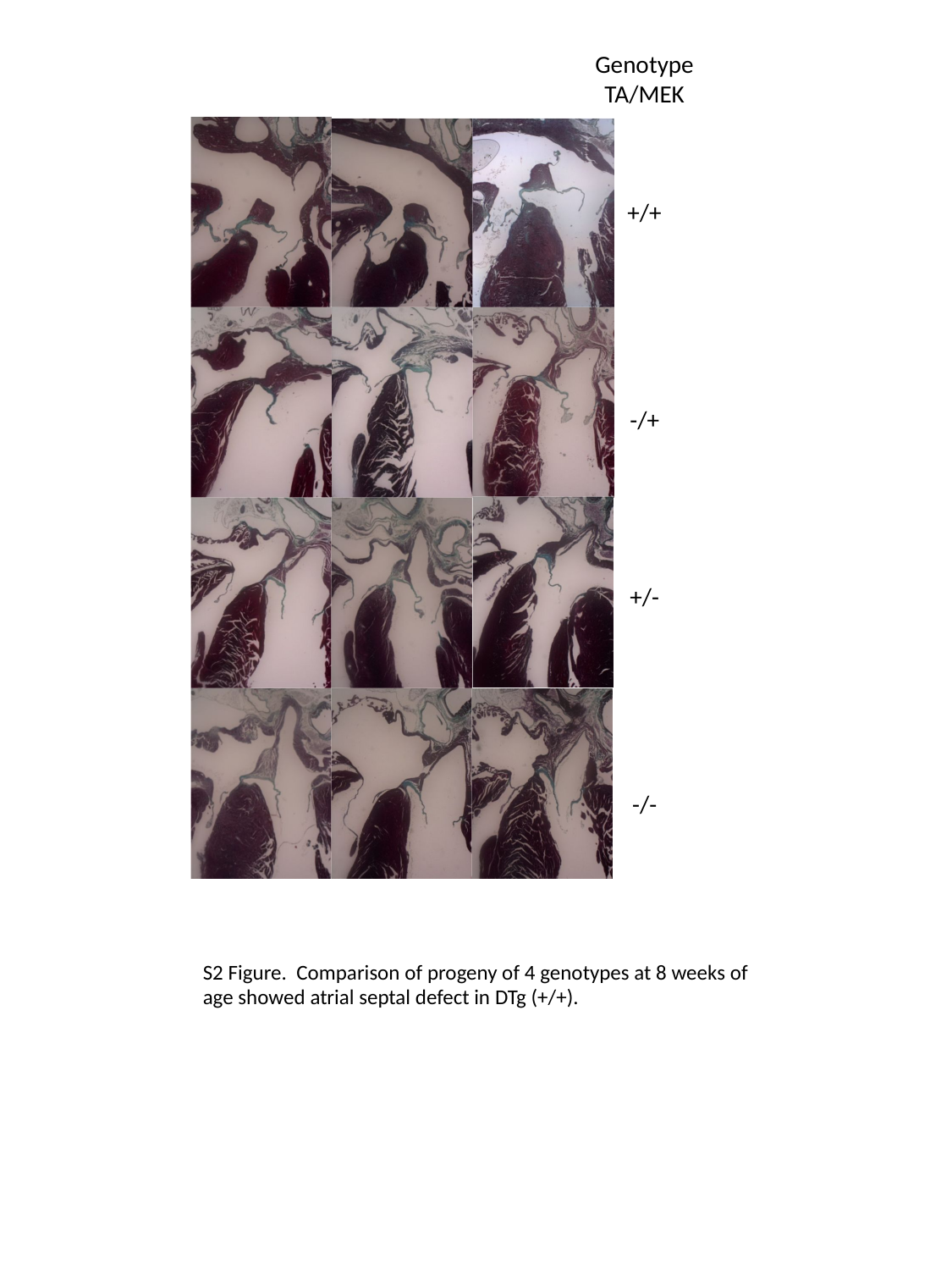

Genotype
TA/MEK
+/+
-/+
+/-
-/-
S2 Figure. Comparison of progeny of 4 genotypes at 8 weeks of age showed atrial septal defect in DTg (+/+).
