# Supplementary figures and images for: Atrial ERK1/2 activation in the embryo leads to incomplete Septal closure: a novel mouse model of atrial Septal defect
Source: J Biomed Sci. 2017 Nov 24;24:89. doi: 10.1186/s12929-017-0392-2 (PMC5702213; doi:10.1186/s12929-017-0392-2)

## Slide 1
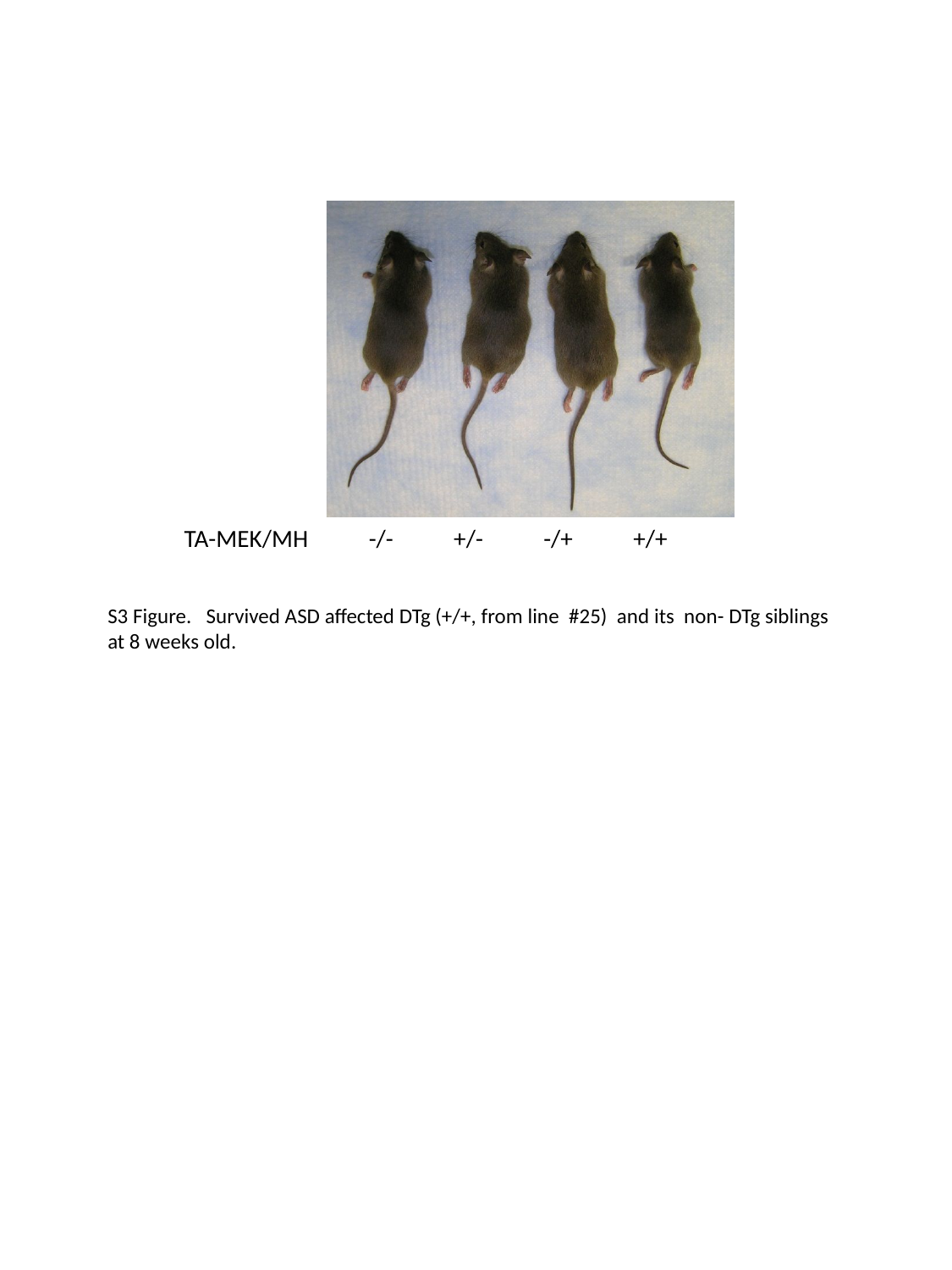

TA-MEK/MH -/- +/- -/+ +/+
S3 Figure. Survived ASD affected DTg (+/+, from line #25) and its non- DTg siblings at 8 weeks old.

Supplement: Supplementary file 3 — S3 Figure. Survived ASD affected DTg (+/+, from line #25) and its non- DTg siblings at 8 weeks old. (PPTX 206 kb) [file 12929_2017_392_MOESM3_ESM.pptx]

## Slide 1
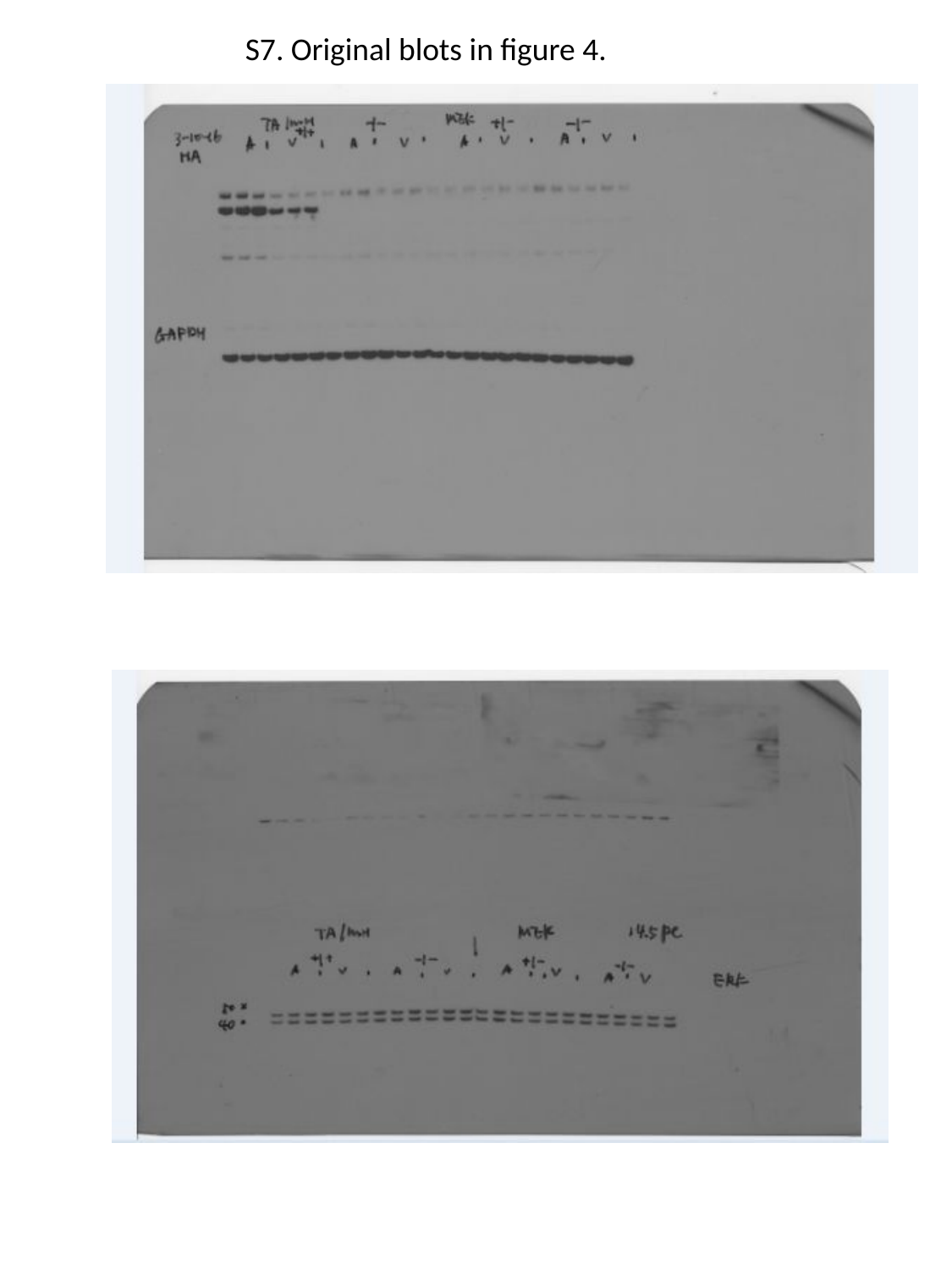

S7. Original blots in figure 4.

## Slide 2
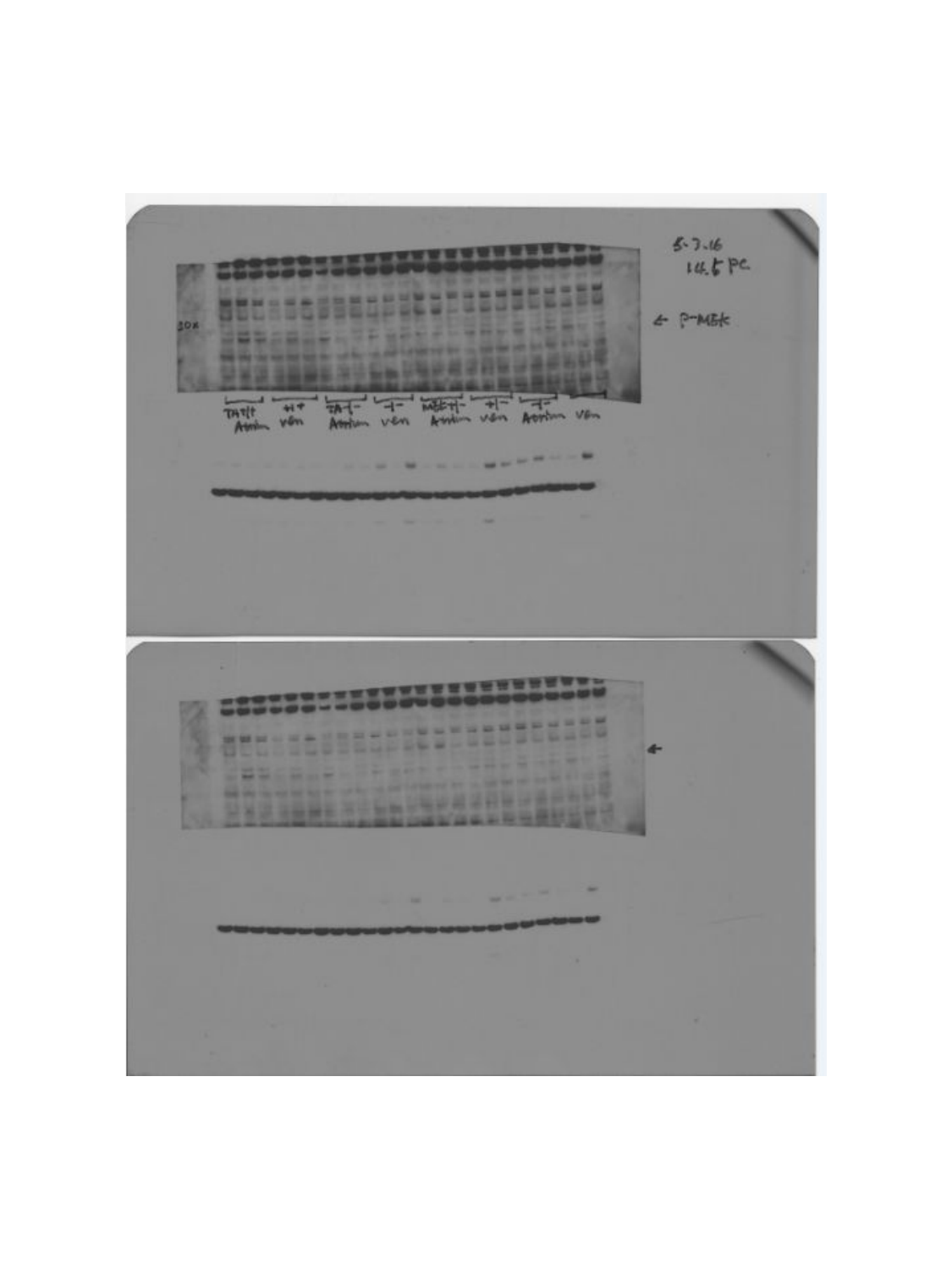

## Slide 3
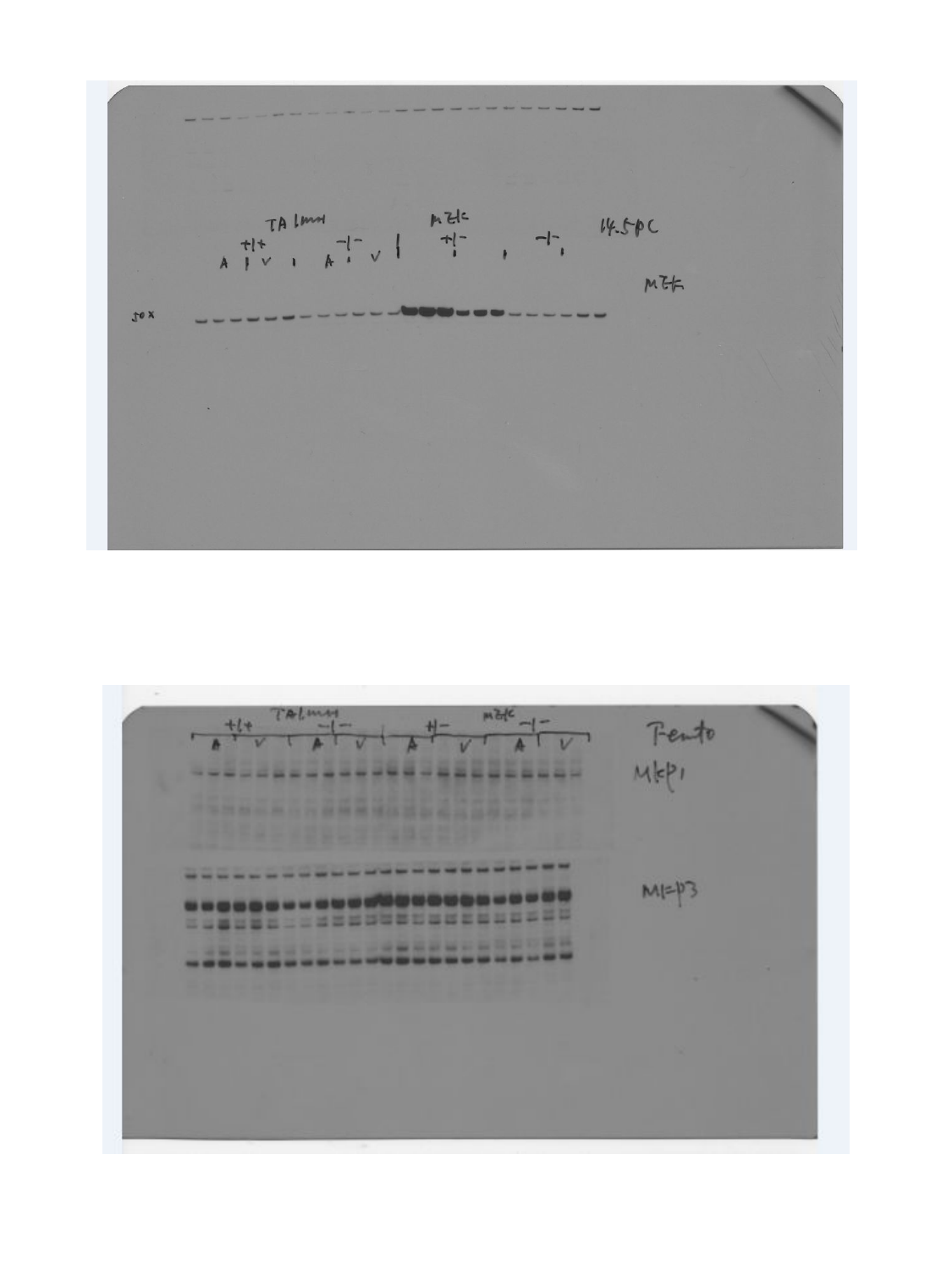

Supplement: Supplementary file 7 — S7. Original blots in fig. 4. (PPTX 1028 kb) [file 12929_2017_392_MOESM7_ESM.pptx]
