# Supplementary material for: Atrial ERK1/2 activation in the embryo leads to incomplete Septal closure: a novel mouse model of atrial Septal defect
Source: J Biomed Sci. 2017 Nov 24;24:89. doi: 10.1186/s12929-017-0392-2 (PMC5702213; doi:10.1186/s12929-017-0392-2)
Supplement: Supplementary file 4 — S4 Figure Anatomical dissection revealed ASD in two independent DTg lines at 8 weeks old. A. line #25, derived from expression sequence using pTet-Splice vector; B. line #8, derived from expression sequence using pTREtight vector. C. MEK1/2, P-ERK1/2, ERK1/2, MKP1, and MKP3 protein levels as detected by Western blot in atrial (A) and ventricular (V) tissue from 14.5 dpc DTg (+/+) and WT (−/−) littermates from line #8 (C57BI/6-FVBN mixed background) and MKP1, and MKP3 from control pure C57BI/6 mice. D. Survival among DTg mice from line #8 with (n = 11) and without (n = 12) ASD, and among non-DTg littermates (n = 66). (PDF 1225 kb) [file 12929_2017_392_MOESM4_ESM.pdf]

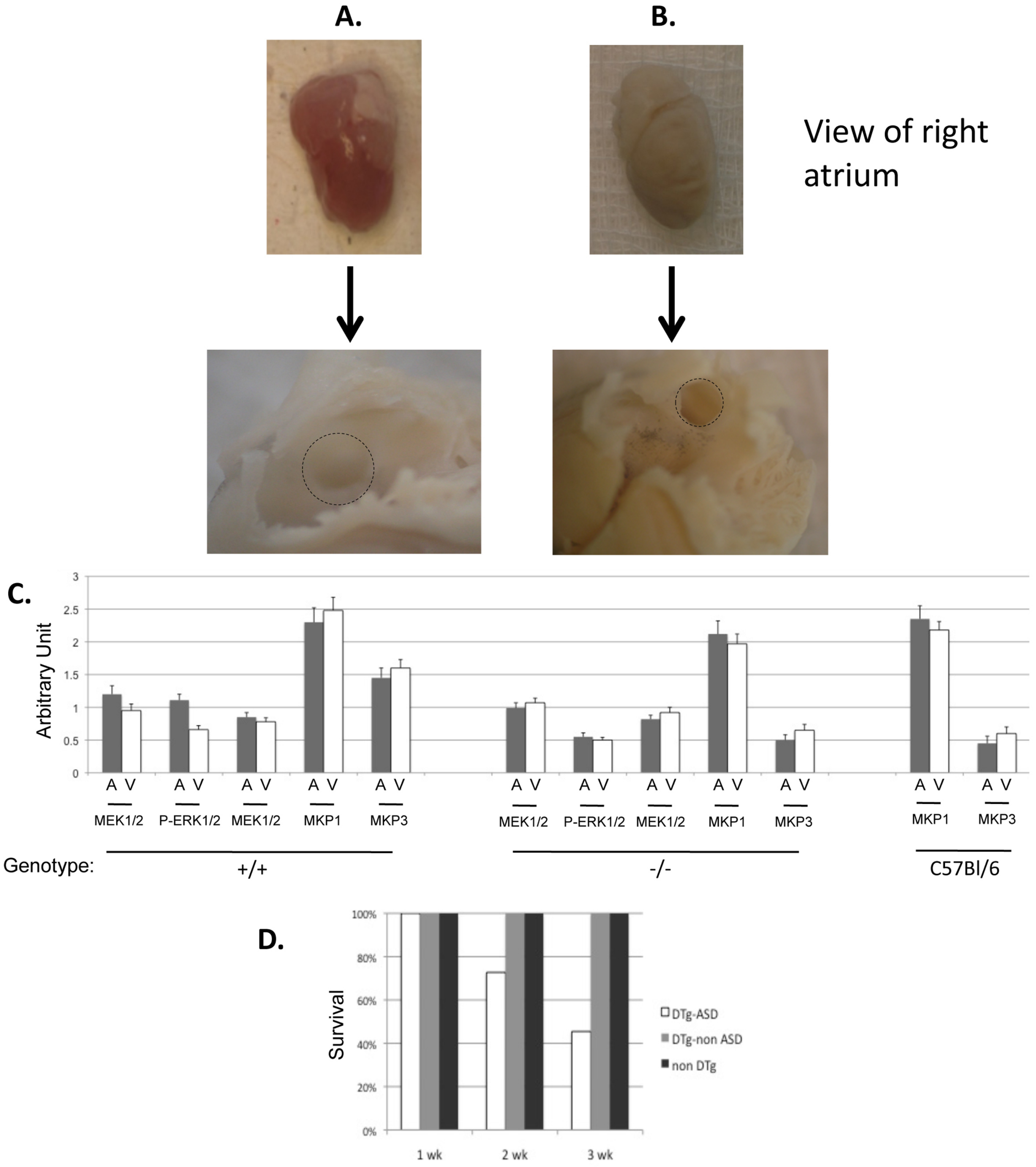

S4 Figure. Anatomical dissection revealed ASD in two independent DTg lines at 8 weeks old. A. line #25, derived from expression sequence using pTet-Splice vector; B. line #8, derived from expression sequence using pTREtight vector. C. MEK1/2, P-ERK1/2, ERK1/2, MKP1, and MKP3 protein levels as detected by Western blot in atrial (A) and ventricular (V) tissue from 14.5 dpc DTg (+/+) and WT (-/-) littermates from line #8 (C57Bl/6-FVBN mixed background) and MKP1, and MKP3 from control pure C57Bl/6 mice. D. Survival among DTg mice from line #8 with (n=11) and without (n=12) ASD, and among non-DTg littermates (n = 66).
