# Supplementary material for: Atrial ERK1/2 activation in the embryo leads to incomplete Septal closure: a novel mouse model of atrial Septal defect
Source: J Biomed Sci. 2017 Nov 24;24:89. doi: 10.1186/s12929-017-0392-2 (PMC5702213; doi:10.1186/s12929-017-0392-2)
Supplement: Supplementary file 5 — S5. Original blots in fig. 1. (PPTX 1641 kb) [file 12929_2017_392_MOESM5_ESM.pptx]

## Slide 1
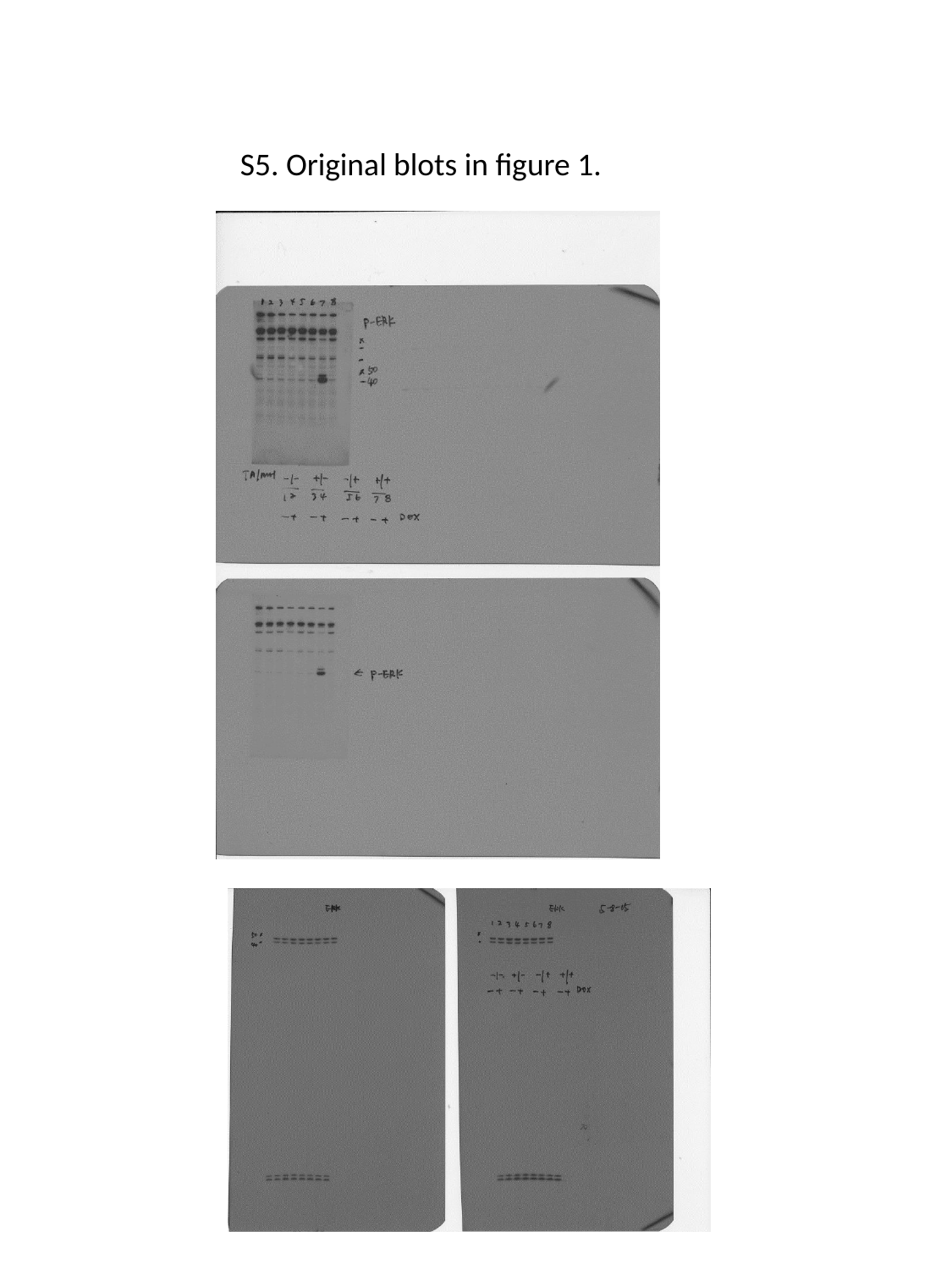

S5. Original blots in figure 1.

## Slide 2
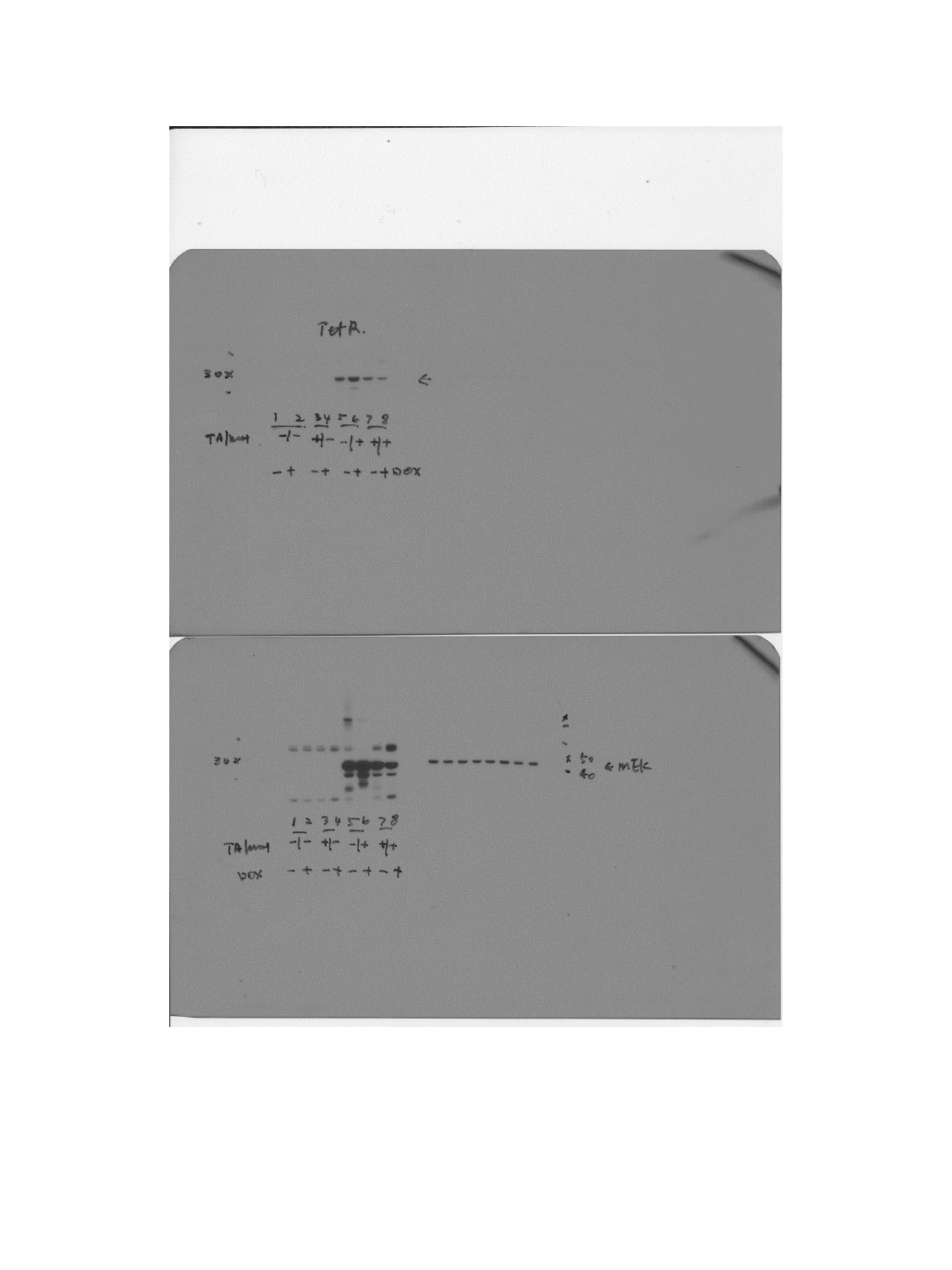

## Slide 3
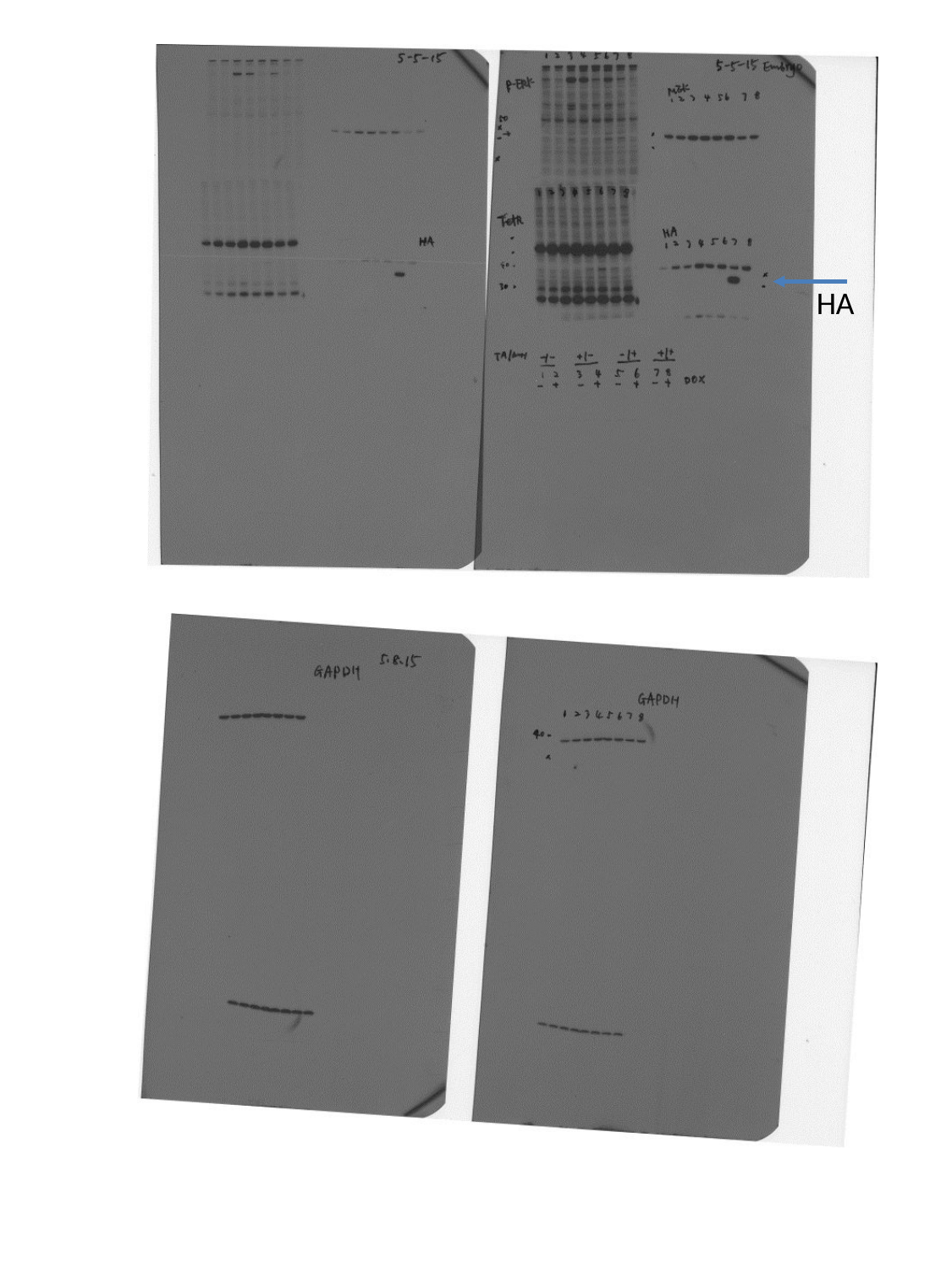

HA

## Slide 4
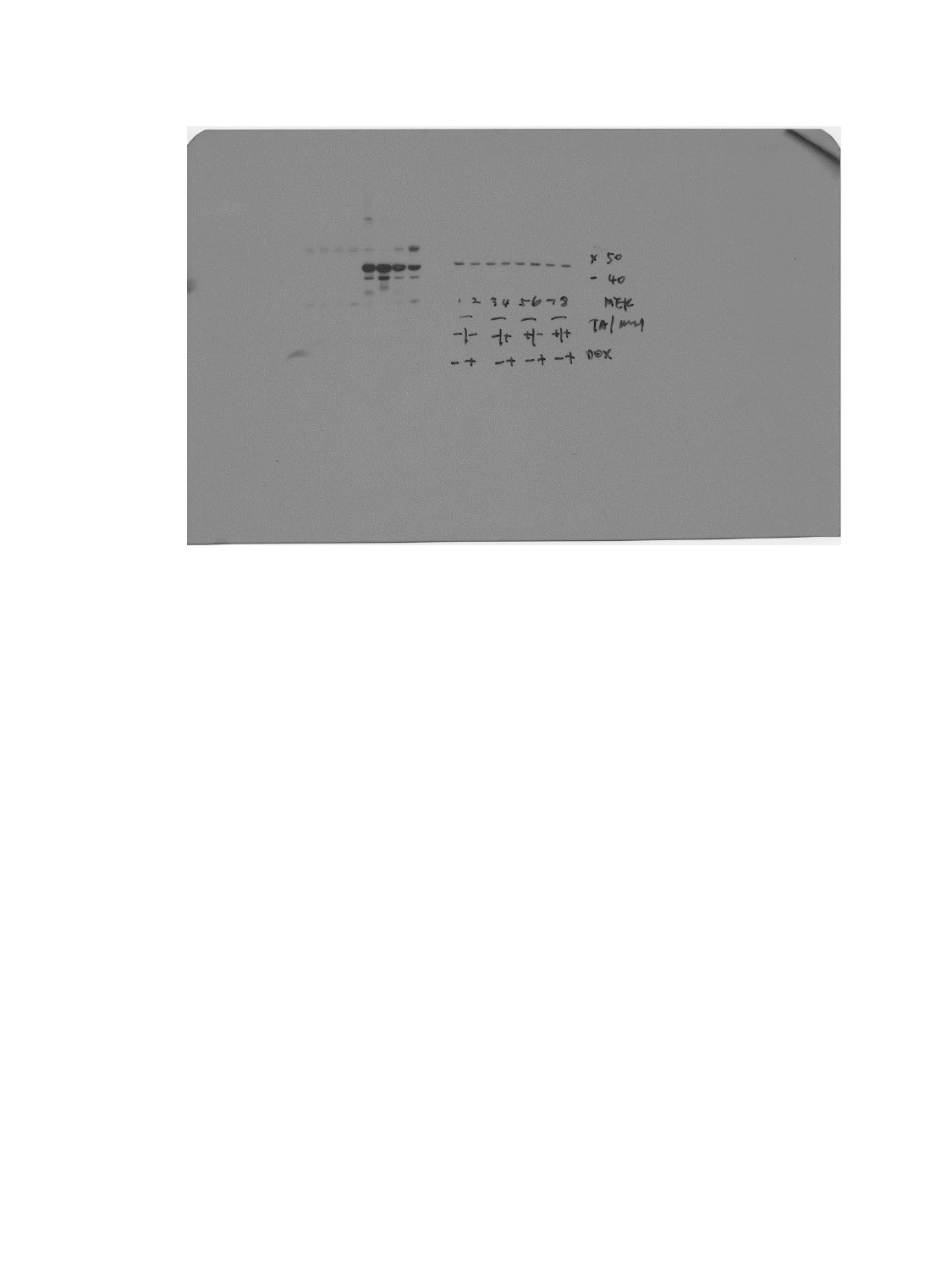

## Slide 5
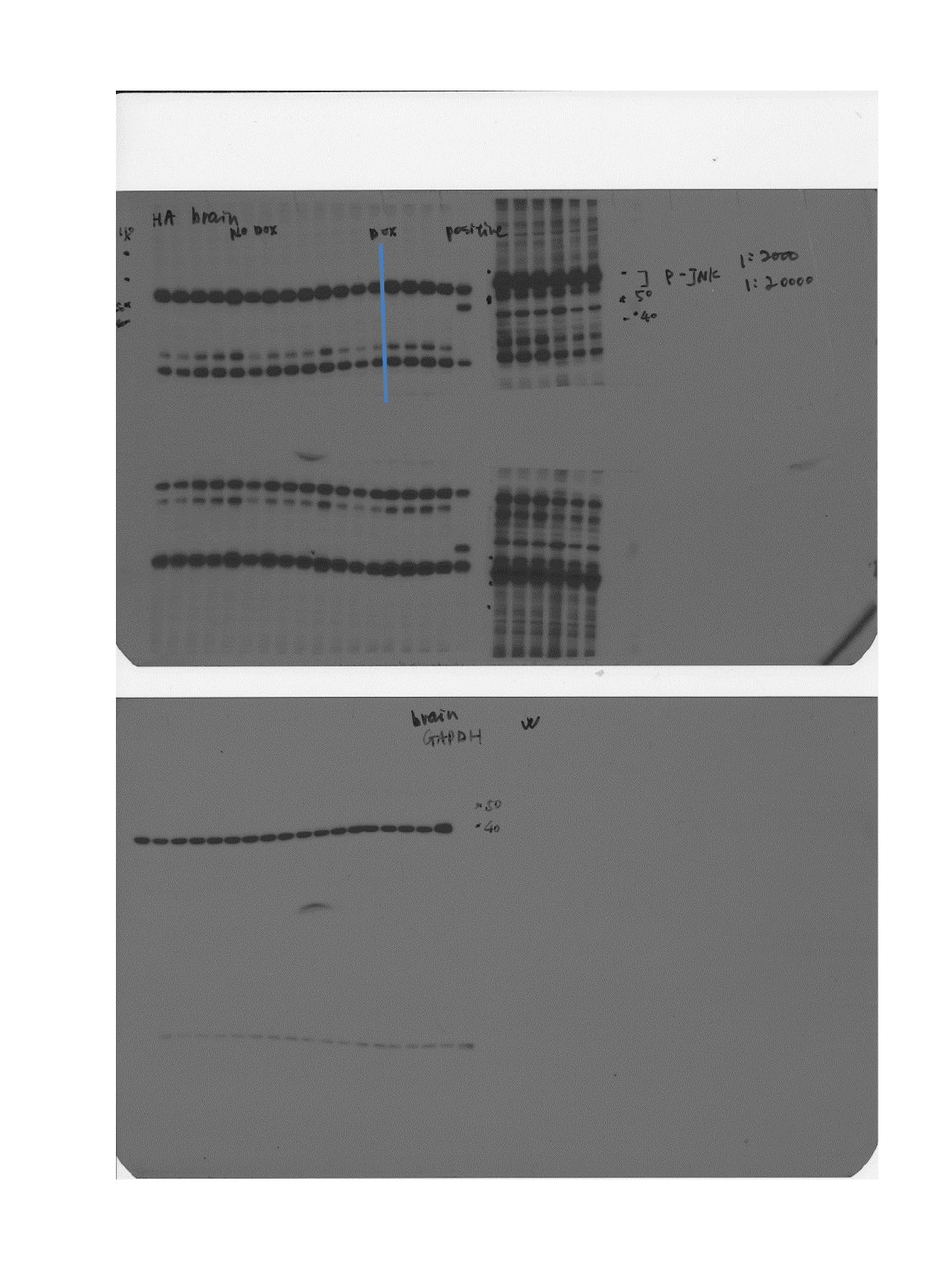

## Slide 6
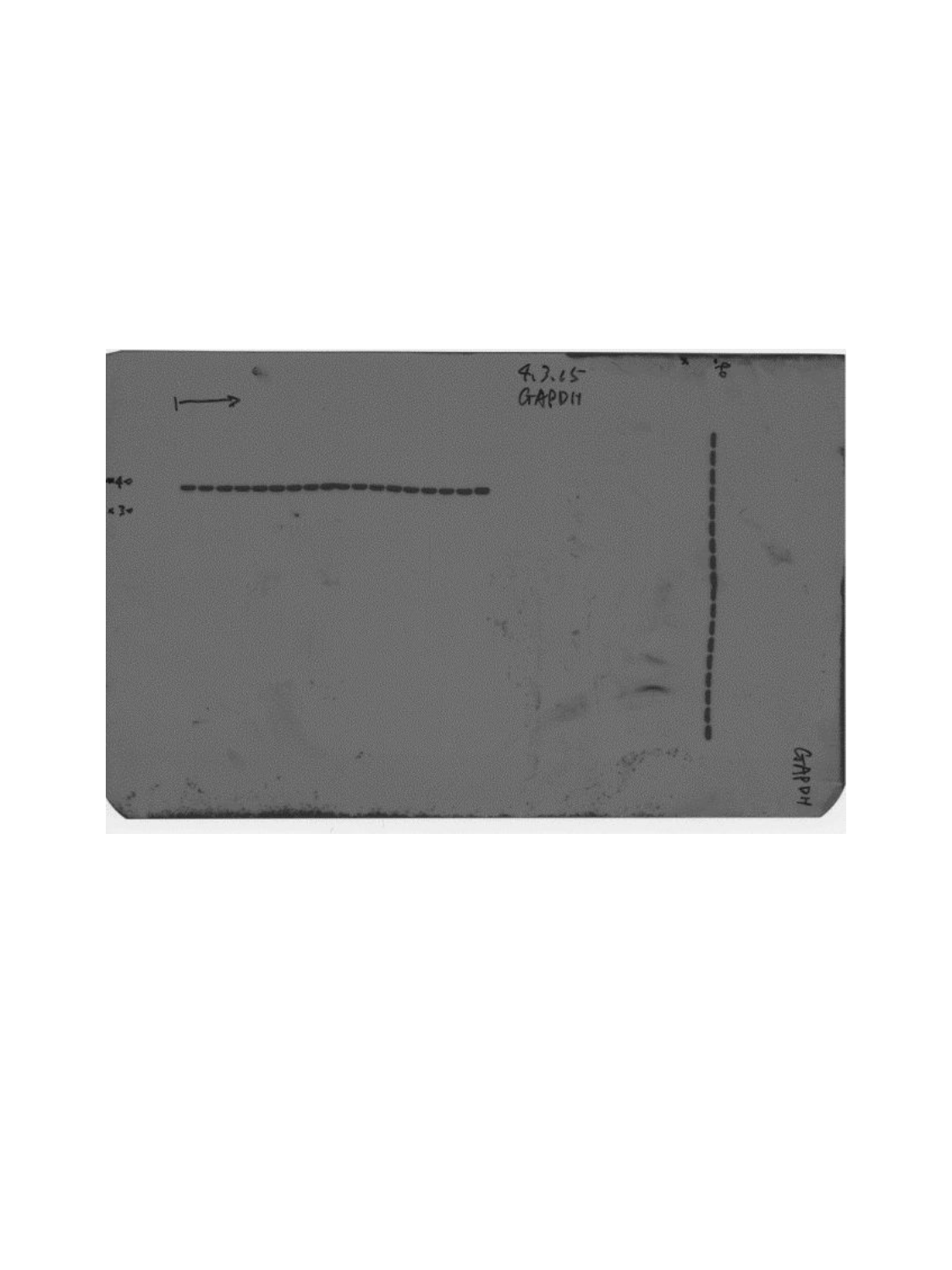

## Slide 7
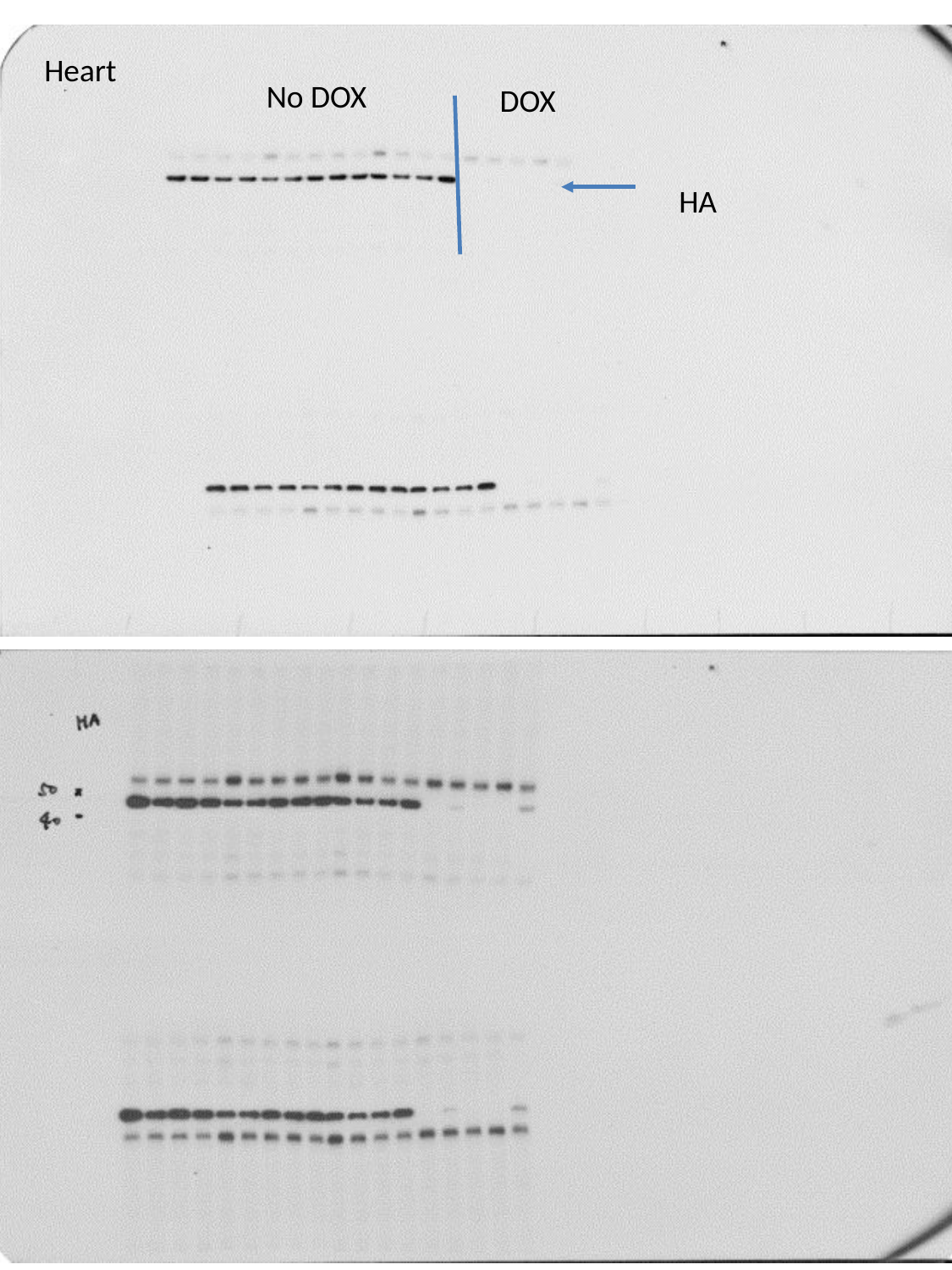

Heart
No DOX
DOX
HA

## Slide 8
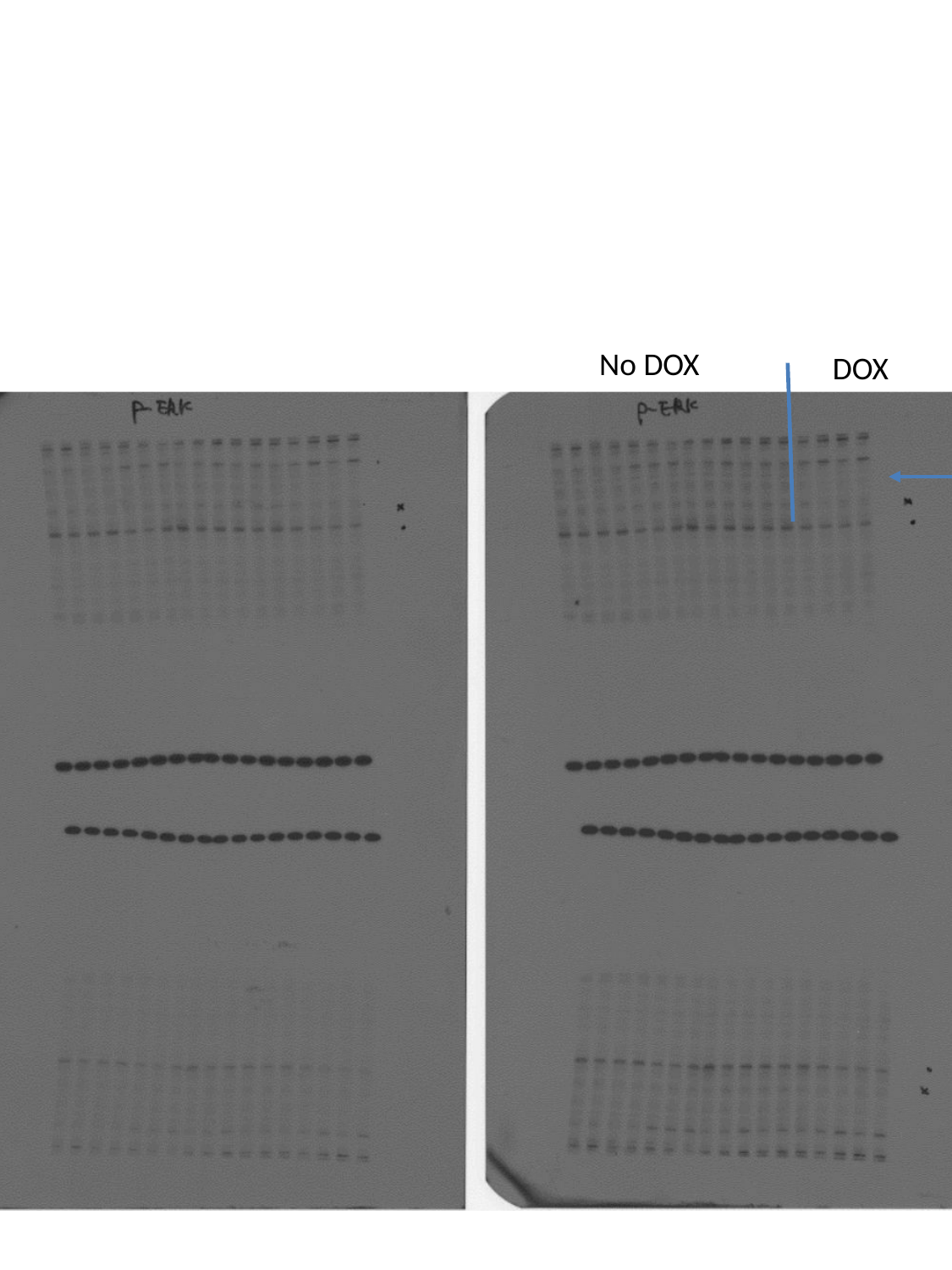

No DOX
DOX
P-ERK

## Slide 9
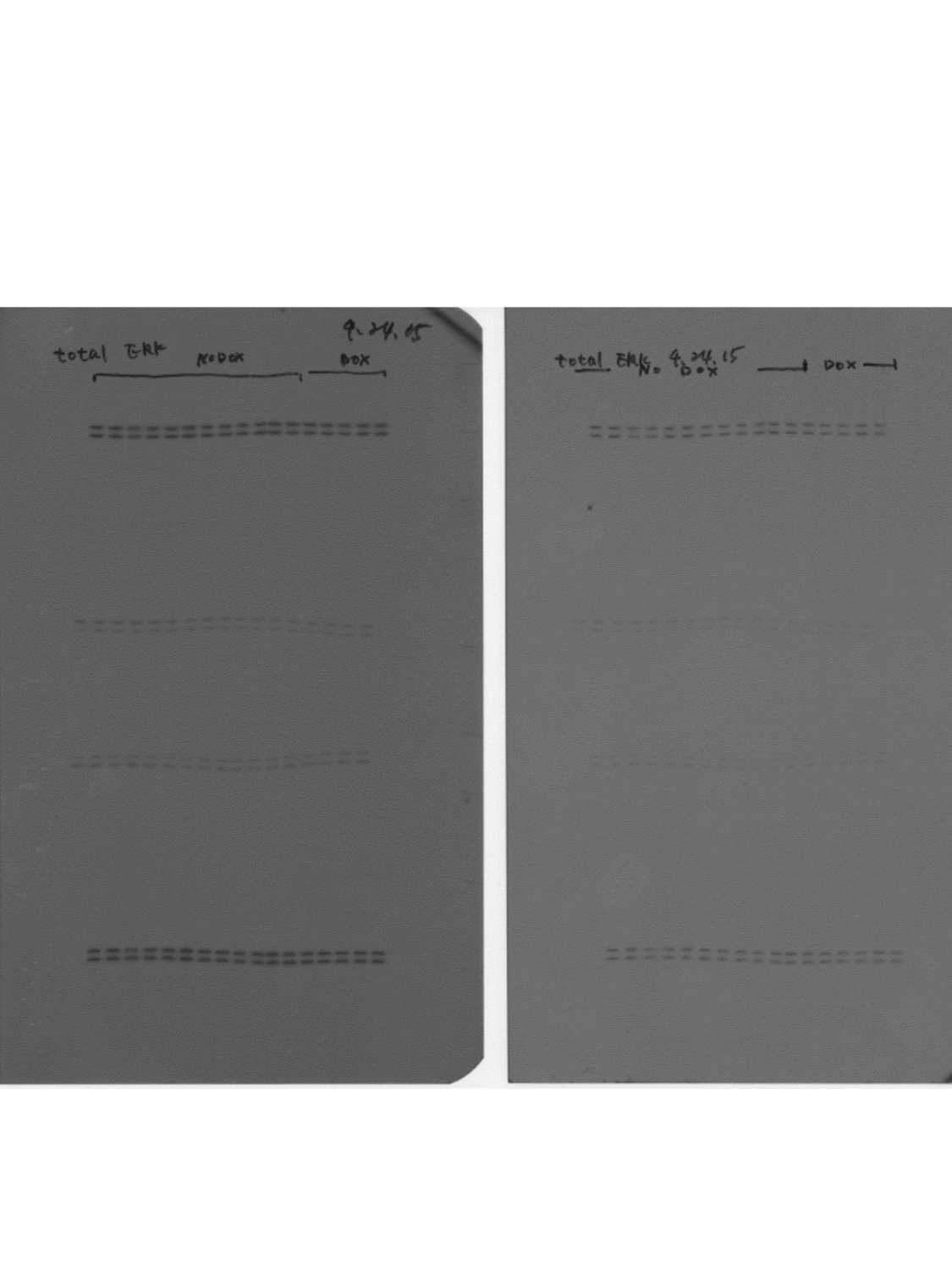

## Slide 10
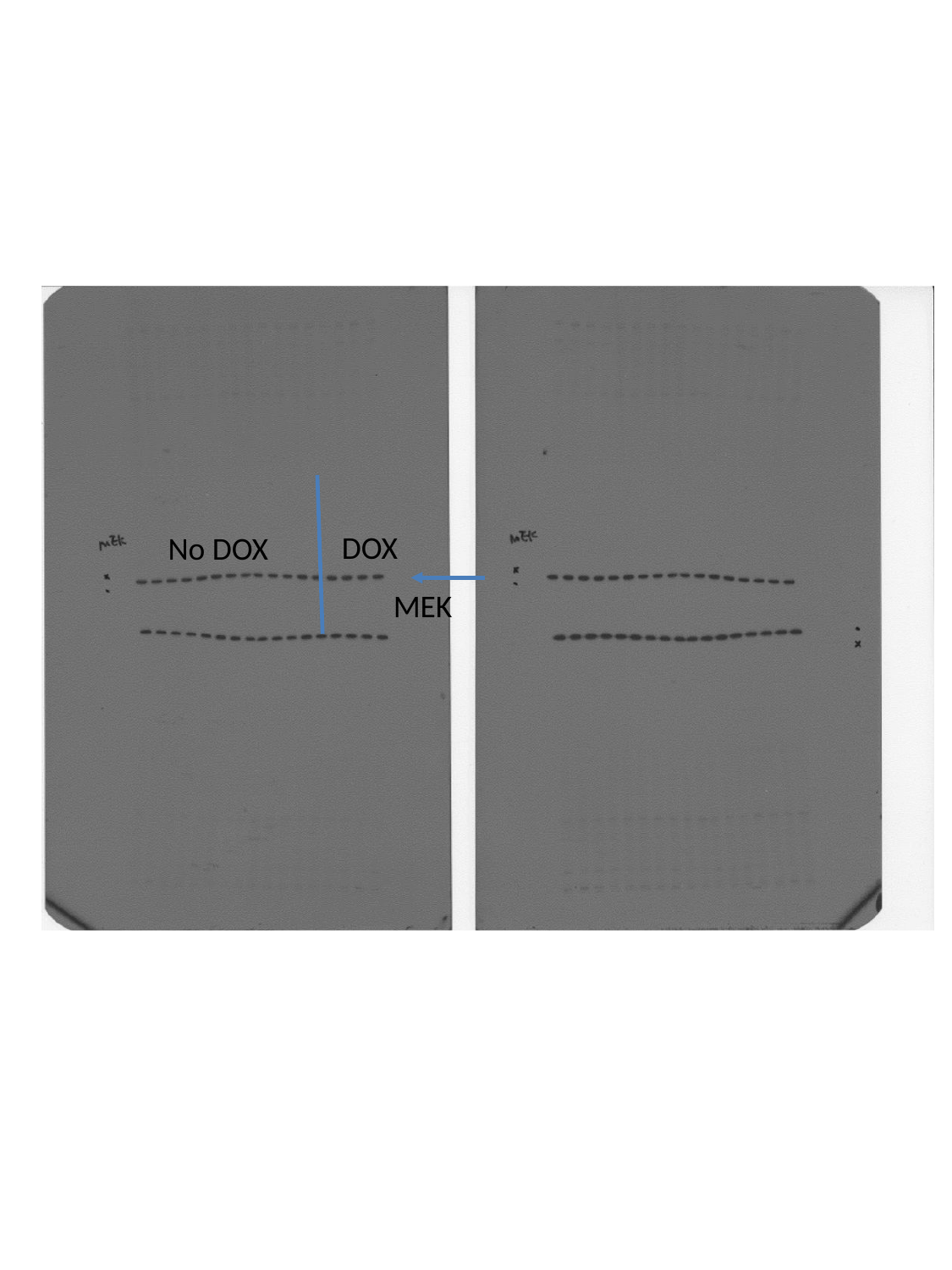

DOX
No DOX
MEK

## Slide 11
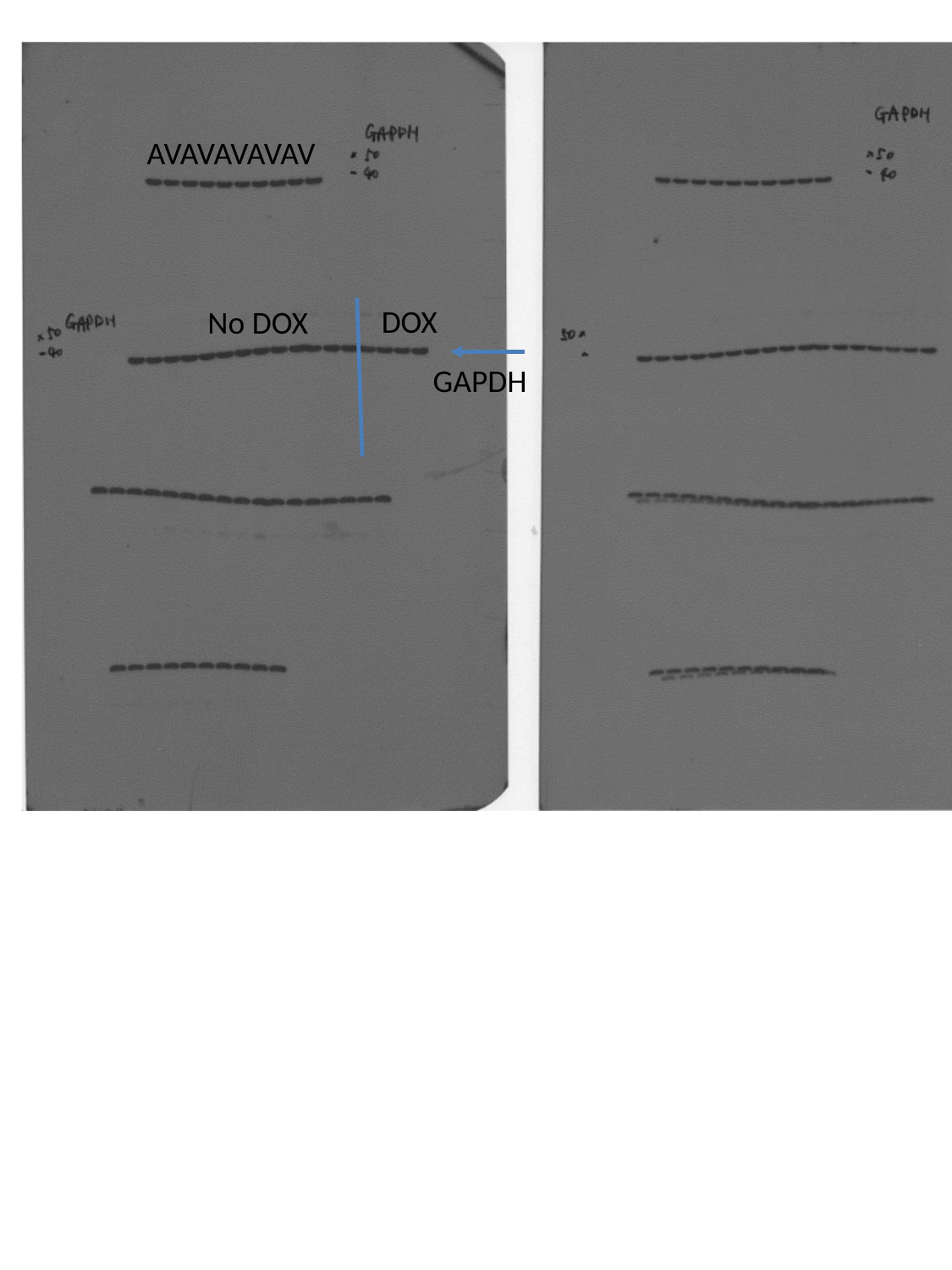

AVAVAVAVAV
DOX
No DOX
GAPDH

## Slide 12
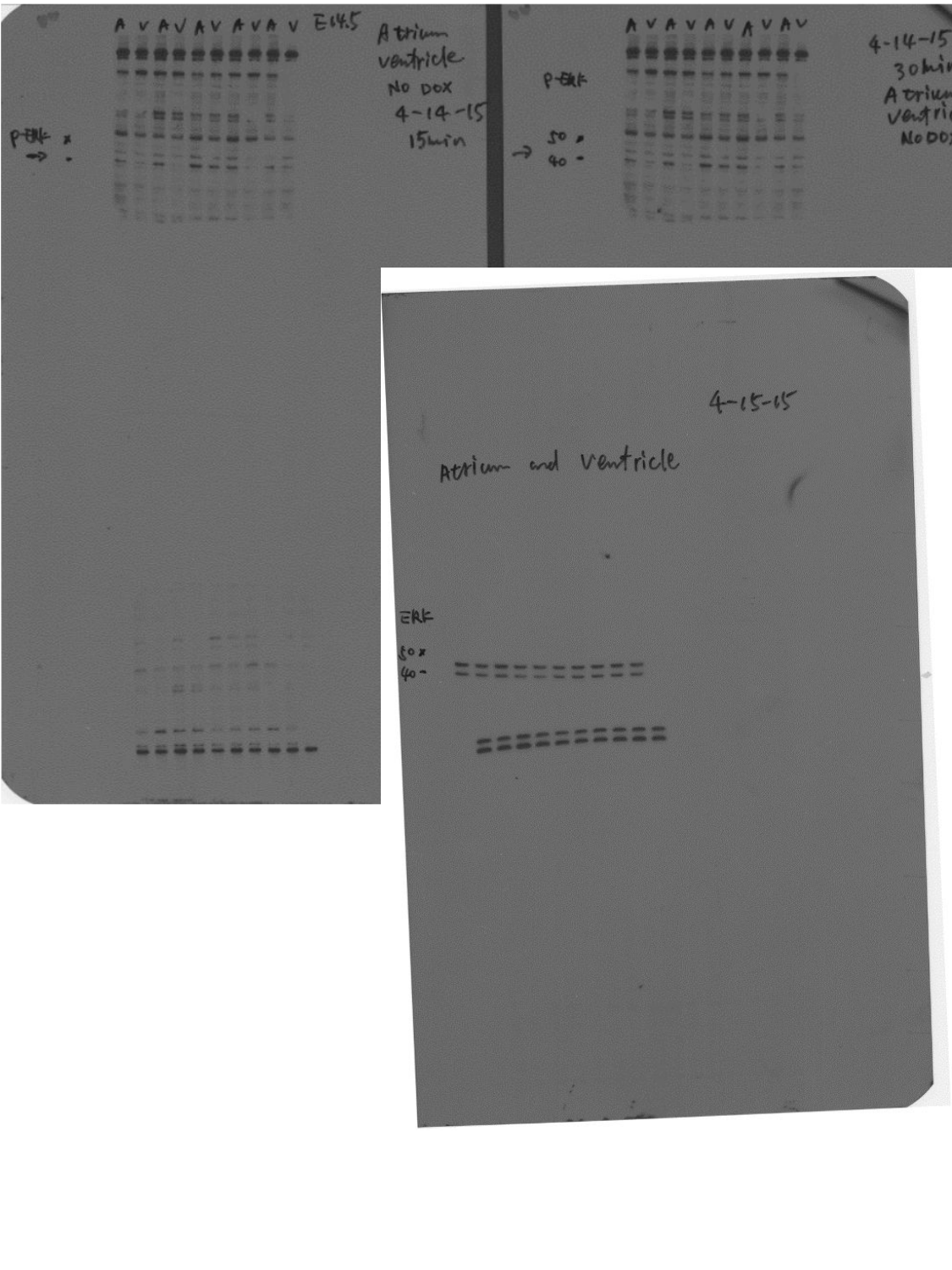

## Slide 13
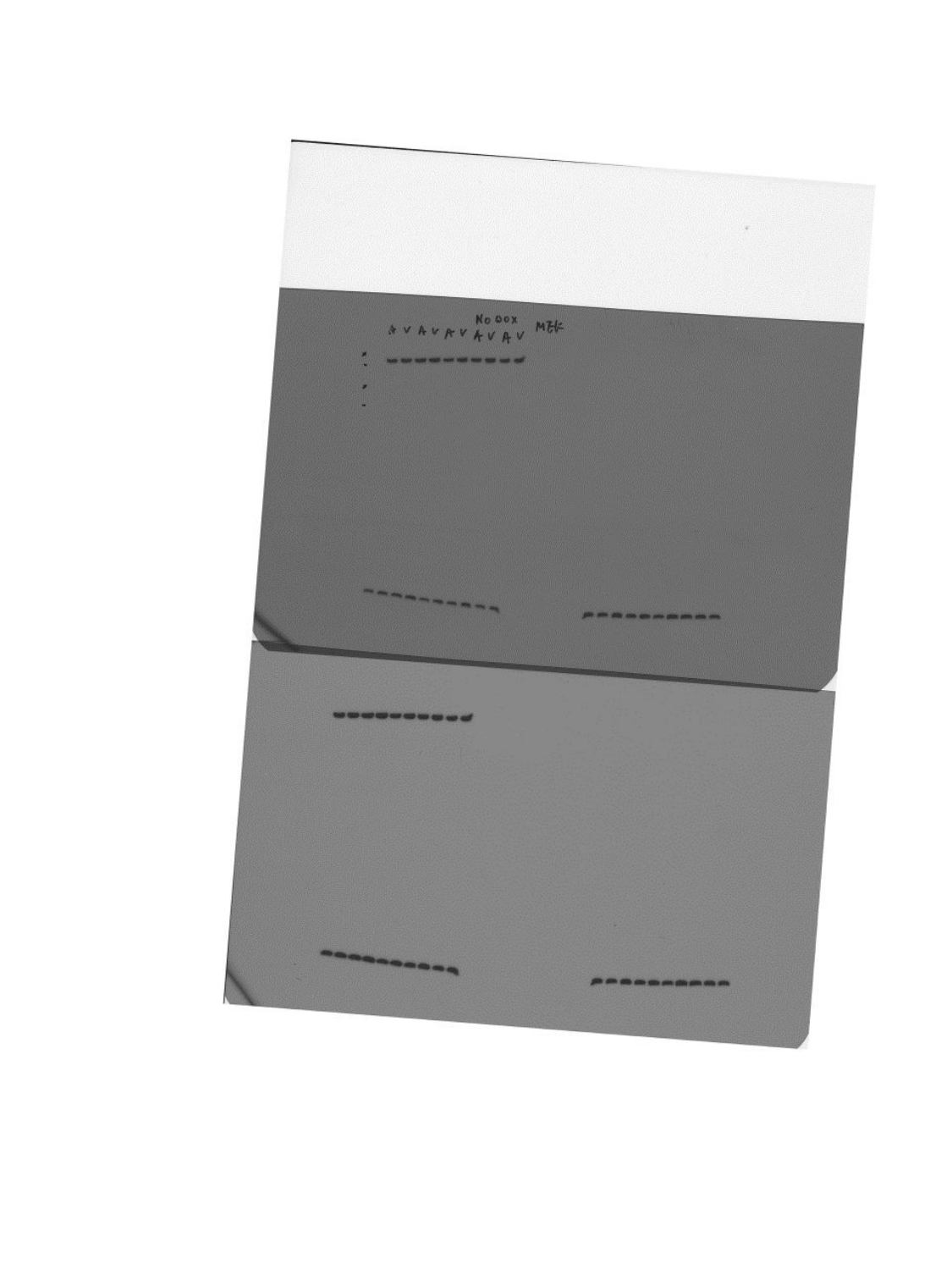

## Slide 14
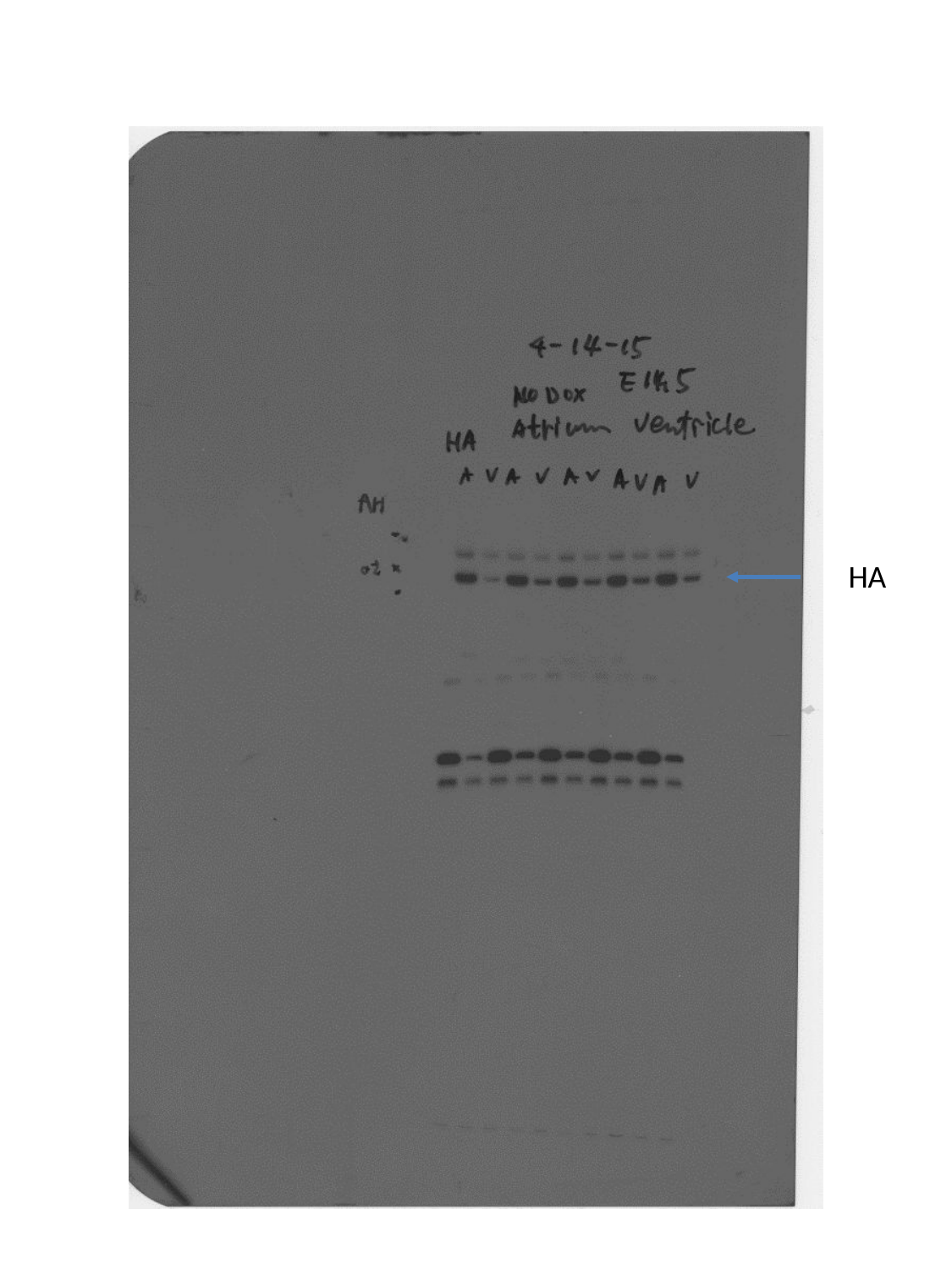

HA
