# Supplementary material for: Atrial ERK1/2 activation in the embryo leads to incomplete Septal closure: a novel mouse model of atrial Septal defect
Source: J Biomed Sci. 2017 Nov 24;24:89. doi: 10.1186/s12929-017-0392-2 (PMC5702213; doi:10.1186/s12929-017-0392-2)
Supplement: Supplementary file 6 — S6 Table Echocardiographic measurements of LV function in the 8 weeks old DTg mice (ASD and non-ASD). (DOCX 14 kb) [file 12929_2017_392_MOESM6_ESM.docx]

**S6. Table Echocardiographic measurements of LV function in the 8 weeks old DTg mice (ASD and non-ASD).**

|  | Non-ASD | ASD |
| --- | --- | --- |
| BW(g) | 21±1 | 22±0 |
| EF(%) | 75±0 | 64±2* |
| FS(%) | 47±1 | 37±2* |
| n | 5 | 5 |

BW, body weight; EF, ejection fraction; FS, fractional shortening; +/+ group includes ASD mice only. **P*< 0.05
